# Supplementary material for: Experience of Kenyan researchers and policy-makers with knowledge translation during COVID-19: a qualitative interview study
Source: BMJ Open. 2022 Jun 1;12(6):e059501. doi: 10.1136/bmjopen-2021-059501 (PMC9160583; doi:10.1136/bmjopen-2021-059501)
Supplement: Author's manuscript [file bmjopen-2021-059501.draft_revisions.pdf]

BMJ Open is committed to open peer review. As part of this commitment we make the peer review history of every article we publish publicly available.

When an article is published we post the peer reviewers' comments and the authors' responses online. We also post the versions of the paper that were used during peer review. These are the versions that the peer review comments apply to.

The versions of the paper that follow are the versions that were submitted during the peer review process. They are not the versions of record or the final published versions. They should not be cited or distributed as the published version of this manuscript.

BMJ Open is an open access journal and the full, final, typeset and author-corrected version of record of the manuscript is available on our site with no access controls, subscription charges or pay-per-view fees (http://bmjopen.bmj.com).

If you have any questions on BMJ Open's open peer review process please email info.bmjopen@bmj.com

BMJ Open

Knowledge translation during COVID-19: Experience of Kenyan researchers and policymakers

Journal:	*BMJ Open*
Manuscript ID	bmjopen-2021-059501
Article Type:	Original research
Date Submitted by the Author:	26-Nov-2021

Complete List of Authors:	Guleid, Fatuma; KEMRI-Wellcome Trust Research Programme Nairobi,<br>Njeru, Alex; KEMRI-Wellcome Trust Research Programme Nairobi<br>Kiptim, Joy; KEMRI-Wellcome Trust Research Programme Nairobi<br>Kamuya, Dorcas; KEMRI-Wellcome Trust Research Programme, Health Systems and Research Ethics; University of Oxford, Nuffield Department of medicine<br>Okiro, Emelda ; KEMRI-Wellcome Trust Research Programme Nairobi, Population Health ; University of Oxford, Nuffield department of medicine<br>Tsofa, Benjamin; KEMRI-Wellcome Trust Research Programme, Health Policy and Systems Research<br>English, Mike; KEMRI-Wellcome Trust Research Programme Nairobi, Health Services Unit; University of Oxford, Nuffield Department of Medicine<br>Molyneux, Sassy ; KEMRI-Wellcome Trust Research Programme, Health Systems and Research Ethics; University of Oxford, Nuffield Department of Medicine<br>Kariuki, David; Ministry of Health, Department of Health Policy and Research<br>Barasa, Edwine; KEMRI-Wellcome Trust Research Programme Nairobi, Health Economics Research Unit; University of Oxford, Nuffield Department of Medicine

Keywords:	COVID-19, Health policy < HEALTH SERVICES ADMINISTRATION & MANAGEMENT, Public health < INFECTIOUS DISEASES

SCHOLARONE™
Manuscripts

I, the Submitting Author has the right to grant and does grant on behalf of all authors of the Work (as defined in the below author licence), an exclusive licence and/or a non-exclusive licence for contributions from authors who are: i) UK Crown employees; ii) where BMJ has agreed a CC-BY licence shall apply, and/or iii) in accordance with the terms applicable for US Federal Government officers or employees acting as part of their official duties; on a worldwide, perpetual, irrevocable, royalty-free basis to BMJ Publishing Group Ltd ("BMJ") its licensees and where the relevant Journal is co-owned by BMJ to the co-owners of the Journal, to publish the Work in this journal and any other BMJ products and to exploit all rights, as set out in our licence.

The Submitting Author accepts and understands that any supply made under these terms is made by BMJ to the Submitting Author unless you are acting as an employee on behalf of your employer or a postgraduate student of an affiliated institution which is paying any applicable article publishing charge ("APC") for Open Access articles. Where the Submitting Author wishes to make the Work available on an Open Access basis (and intends to pay the relevant APC), the terms of reuse of such Open Access shall be governed by a Creative Commons licence – details of these licences and which Creative Commons licence will apply to this Work are set out in our licence referred to above.

Other than as permitted in any relevant BMJ Author's Self Archiving Policies, I confirm this Work has not been accepted for publication elsewhere, is not being considered for publication elsewhere and does not duplicate material already published. I confirm all authors consent to publication of this Work and authorise the granting of this licence.

For peer review only

Knowledge translation during COVID-19: Experience of Kenyan researchers and policymakers

Fatuma H Guleid[1], Alex Njeru[1], Joy Kiptim[1], Dorcas Kamuya[2,7], Emelda Okiro[3,7], Benjamin Tsofa[2], Mike English[4,7], Sassy Molyneux[2,7], David Kariuki[6], Edwine Barasa[5,7]

1. Policy Engagement & Knowledge Translation Unit, KEMRI-Wellcome Trust Research Programme, Kenya
2. Health Systems & Research Ethics Department, KEMRI-Wellcome Trust Research Programme, Kenya
3. Population Health Unit, KEMRI-Wellcome Trust Research Programme, Kenya
4. Health Services Unit, KEMRI-Wellcome Trust Research Programme, Kenya
5. Health Economics Research Unit, KEMRI-Wellcome Trust Research Programme, Kenya
6. Department of Health Policy and Research, Ministry of Health, Kenya
7. Center for Tropical Medicine and Global Health, Nuffield Department of Medicine, University of Oxford, Oxford, UK

Abstract

Objective Researchers at the KEMRI-Wellcome Trust Research programme (KWTRP) carried out Knowledge Translation (KT) activities to support policymakers as the Kenyan Government responded to the COVID-19 pandemic. We assessed the usefulness of these activities to identify the facilitators and barriers to KT and suggest actions that facilitate KT in similar settings.

Settings and participants Researchers at KWTRP in Kenya who were involved in KT activities during the COVID-19 pandemic (n=6) were selected to participate in key informant interviews to describe their experience. In addition, the policymakers with whom these researchers engaged were also invited to participate (n=11). Data was collected from March 2021-August 2021.

Analysis A thematic analysis approach was used using a predetermined framework to develop a coding structure consisting of the core thematic areas. Any other theme that emerged in the coding process was also included.

Results Both groups reported that the KT activities increased evidence availability and accessibility, enhanced policy makers' motivation to use evidence, improved capacity to use research evidence, and strengthened relationships. Policymakers shared that a key facilitator of this was the knowledge products shared and the regular interaction with researchers. Both groups mentioned a key barrier was the timeliness of generating evidence, which was further exacerbated by the pandemic context. Thus, both groups felt it was important to institutionalise KT to improve readiness to respond to public health crises.

Conclusion This study provides a real-world example of the use of KT during a public health crisis. It further highlights the need to fully embed KT within the core functions of research and health policy institutions to build on the structures that support KT initiatives that readily respond to public health emergencies.

For peer review only - http://bmjopen.bmj.com/site/about/guidelines.xhtml

Strength and limitations of this Study

- This study provides an example of how KT can be used in Low/middle income countries to support decision making during health emergencies.
- Some researchers who participated in the interviews were also part of the study team which introduces bias. However, the results of the study were internally and externally shared to check for objectivity and to ensure the views presented here were an accurate representation of researcher and policy makers' views.

Introduction

The COVID-19 pandemic has required policymakers to make decisions that have significant health, social and economic consequences. Incorporating the best available research evidence to guide decision-making is essential for an effective response. However, the rapidly increasing volume of COVID-19 evidence, coupled with the prevalence of misinformation, could overwhelm policymakers striving to use evidence.[1,2].

Knowledge translation (KT) is best placed to respond to this challenge[1,3]. KT broadly involves the synthesis, dissemination, and application of evidence[4]. Researchers who conduct KT play an important role in supporting pandemic responses. However, even in non-emergency settings, there are several documented barriers to KT for both researchers and policymakers. For researchers, these include lack of capacity to plan and implement KT, poor relationships between researchers and policymakers, competing demands for time, a lack of institutional incentives to participate in KT, the complexity of the policy process, and difficulty in communicating research evidence[5-7]. For policymakers, the most frequent barriers include lack of accessible evidence, lack of timely research output, and poor capacity to appraise and understand research evidence[8].

Enhancing Evidence-Informed Decision Making for health (EEVIDEM)

The EEVIDEM project was a KEMRI-Wellcome Trust Research Programme (KWTRP) initiative that sought to promote evidence-informed decision-making in Kenya using four interventions. The first intervention is researcher embeddedness which involves getting KWTRP researchers into policymaking spaces like technical working groups. The second intervention is reciprocal secondment, where a policymaker from the Ministry of Health (MoH) spends 50% of their working hours at KWTRP, allowing them to be involved in research activities; likewise, one researcher from KWTRP spends 50% of their time at the MoH to understand the policymaking space. The third intervention is hosting regular policy dialogues to provide a platform stakeholders to reflect critically on policy issues. The fourth intervention is knowledge synthesis and dissemination (hereafter collectively referred to as "KT activities"). This intervention involved generating evidence and developing and disseminating KT products to policymakers and is the paper's focus.

We sought to describe the experiences of the researchers and policymakers involved in KT activities that support the COVID-19 response and assess the influence of these activities. Understanding the researchers' and policy makers' views helps address the

For peer review only - http://bmjopen.bmj.com/site/about/guidelines.xhtml

factors affecting evidence use to promote evidence uptake as the pandemic evolves and strengthen KT initiatives in the future.

Methods

Study setting

In Kenya, the Ministry of Health (MoH) has established organisational and institutional structures to support using evidence in policymaking. The Kenya Medical Research Institute (KEMRI), of which KWTRP is part, is mandated by the MoH to generate this evidence. During the pandemic, the MoH established the National COVID-19 Task Force. This task force draws its membership from the MoH and other government agencies, research and academic institutions, United Nations agencies, civil society organisations and the private sector. The task force coordinates and mobilises technical advice to the MoH and advise other ministries on appropriate measures. The implementation of the Task Force's action points are carried out within the MoH's departmental structure, which includes the Director General's Office and the Emergency Operations Centre (EOC). KWTRP researchers provided research evidence and engaged with policymakers within these structures.

EEVIDEM Conceptual framework

To guide the assessment of the influence of KWTRP's KT activities on evidence uptake during the COVID-19 pandemic, we adapted a determinant framework (Fig. 1) that conceptualises determinants that have been found to influence KT outcomes[8-10]. This framework emphasises four overlapping and interacting determinants, namely (1) availability and accessibility of evidence; (2) attitude/motivation to search for and use evidence; (3) capacity to appraise and utilise evidence; and (4) the relationship between the researchers and policymakers.

KT activities

Initially, the researchers synthesised evidence on questions they thought were of high priority. As the pandemic progressed, policymakers started directly requesting COVID-19 research evidence. The evidence was packaged as rapid response evidence briefs and/evidence summaries. Dissemination was primarily through email, and in some cases, researchers were invited to do oral presentations as a follow-up to explain the evidence further.

Study design

This study adopted a qualitative cross-sectional design. A total of 17 key informant interviews (KIIs) were carried out on two groups: researchers and policymakers (Table 1). All KWTRP researchers involved in KT activities to support the COVID-19 response were interviewed (n=6). The selected researchers identified the policymakers with whom they engaged during their KT activities who were then invited through email to participate in the study. We identified 21 eligible policymakers, and out of this, a total of 11 policymakers were interviewed at which a point of information saturation was reached. EEVIDEM's conceptual framework was used to develop the interview guides and to facilitate the interviews. Separate guides were used for researchers and policymakers. All the interviews were conducted virtually using Microsoft Teams by

For peer review only

two researchers (FG and AN) after each participant provided informed consent. Each interview lasted between 30-60 minutes and was audio-recorded using an encrypted audio recorder, except for one policymaker who declined consent to audio recording - the interview was captured by a note-taker in handwritten notes. Data was collected from March to August 2021.

Patients and public involvement

No patients were involved in this study.

Table 1: Institutions and departments of participants in the study

Researchers	Policymakers'
Health Economics Research Unit	Ministry of Health
Health Systems and Policy Research group	Ministry of Health-Emergency operations Center (EOC)
Health Services Research unit	Ministry of Health
Virus Epidemiology and Control Research group	Presidential Policy and Strategy Unit
Virus Epidemiology and Control Research group	Ministry of Health-EOC
Policy Engagement and Knowledge Translation	World Health Organisation-Kenya office
	Ministry of Health-EOC
	Ministry of Health-Office of the Cabinet Administrative Secretary of Health
	Ministry of Health-Office of the Director General of Health
	Ministry of Health-Department of Health Policy & Research
	Kilifi County Department of Health services

Data management and analysis

All audio recordings were transcribed verbatim in MS Word. The transcripts were coded using NVIVO qualitative data analysis software (QSR International Pty Ltd. Version 12, 2014). For analysis, a predetermined coding structure consisting of the

core thematic areas of the conceptual framework was used. Any other theme that emerged in the coding process was also included. Once all data were coded in their respective themes, code reports were generated and analysed (FG & AN).

Ethical considerations

This study received ethical approval from KEMRI's Scientific and Ethics Review Committee (KEMRI/RES/7/3/1). Information sheets detailing the study description were shared with all participants before the interviews, and signed consent was obtained.

Results

Fifty-Six rapid response evidence briefs and summaries were developed. These briefs shared evidence from primary studies, including modelling studies, genomic surveillance of SARS-CoV-2, seroprevalence of SARS-CoV-2 antibodies, health systems assessment and economics studies, and clinical surveillance; and from the synthesis of existing evidence on topics requested by policymakers (Table 2). Briefs were 2-4 pages long and included key messages, brief background, and evidence findings.

Table 2: Summary of briefs shared by researchers

Topic	Number
Seroprevalence and genomic surveillance of SARS-CoV-2	21
Other COVID-19 related briefs	26
Clinical surveillance	9

Participants described several facilitators and barriers that shaped their experience and influenced evidence uptake, as summarised in Table 3.

1. Accessibility and availability

The researchers felt that their KT efforts increased evidence availability and accessibility. A significant facilitator of this was the institutional support and existing KT capacity in KWTRP that provided guidance and supported researchers in the consistent and timely development of KT products.

"*it has been the existence of the structures that we've managed to create with this platform [EEVIDEM]. It's something that we don't emphasise enough, having that pool of people*." KII-R05, Researcher

Policymakers also reported that the briefs enhanced their access to research evidence. They highlighted the format of the briefs as important in making evidence more accessible as the briefs presented evidence in concise and simplified language.

For peer review only

"*I found the format fairly useful. I like that there was a section that was a summary of what the rest of the brief held. It was beneficial as one can quickly garner some of the key evidence from that particular brief very quickly*" KII-PM08, Policymaker

"*It's easy to understand. So, when the person reading understands it well, they're can give the information in the same way without contorting the information*." KII-PM09, Policymaker

Both groups reported the importance of augmenting written briefs with an oral presentation of evidence which provided them with an opportunity to discuss the evidence and reduce misinterpretation:

"*They [the researchers] present most of the information and not only presented in writing, they normally present there [at the MoH]. So, you can always seek clarification should there be a need to.*" KII-PM01, Policymaker

"*the presentations are best because they involve you interacting with the people who are making the decisions or who are going to interpret the evidence. So, you get to communicate far more information, unlike these other modes of communication"* KII-03, Researcher

The main barrier to evidence availability and accessibility for both groups was timeliness. The time it took to generate and synthesise high-quality evidence was often longer than the time within which evidence was needed for timely policymaking.

"*Sometimes they are required urgently, and I have to turn them around while ensuring that they're of reasonable quality which can be a challenge*" KII-R05, Researcher

"*the challenge that is experienced is the length of time from once a request is sent out for a certain matter to the time a brief is submitted on the same*" KII-PM01, Policymaker

Other barriers mentioned by researchers included the limited availability of local data which made it difficult for researchers to adapt evidence to local settings. Another barrier was the lack of a feedback mechanism. Some researchers were unaware of how useful briefs were to policymakers as they rarely got direct feedback:

"*you'd expect some feedback where you're told that this thing makes sense and that often doesn't happen. So, there's no feedback mechanism saying, well, we received your brief, and you know*." KII-R03, Researcher

Policymakers mentioned two more barriers. One was the length of the briefs; they felt that when a brief was long or too technical, it lowered the accessibility of the evidence. In addition, policymakers shared that there was unequal access to evidence amongst them.

"*In my position, getting evidence is not as difficult; however, other officers within the directorate who wish to find out more information find it a bit difficult to access the evidence and are told write officially or show that it comes through like DGs office or PS office so that they can access the evidence."* KII-PM01, Policymaker

Researchers highlighted three factors that could improve evidence availability and accessibility. These included: expanding KT capacity in KWTRP, increasing

For peer review only

availability of local data to researchers to support the contextualisation of evidence to the local setting; and, more feedback from policymakers to help researchers determine if policymakers found evidence easier to use through their KT activities. Suggestions by policymakers included making briefs even easier to read by using more illustrations and tables and depositing briefs on open platforms so that more policymakers have access to evidence.

Motivation/attitude

Both groups reported that regular sharing of briefs enhanced policymaker motivation to seek and use research evidence. A key facilitator was the relevance of the evidence. Motivation was enhanced as long as the evidence answered a priority question:

"*To a certain extent, the larger the crisis, the more the need for the research. However, the endless loop for motivation would be, the need to answer a question at the forefront of policymakers' minds*" KII-PM03, Policymaker

A researcher echoed this sentiment:

"*I think when we don't produce outputs that align with their needs, their motivation goes down*." KII-R06, Researcher

Both groups felt that the availability and willingness of researchers to generate and share evidence with policymakers was an important motivator.

"*I think just knowing that we are available to them is a huge motivator. That someone else can search for evidence and summarise it and then present it to them is a huge motivator*." KII-R06, Researcher

"*There are people [researchers] we easily discuss with, and they are available. So, yes, I think they are motivational*." KII-PM01, Policymaker

Policymakers mentioned other facilitators, including the positive feedback loop generated when evidence-informed policies were implemented and prior engagement between the two groups. These factors enhanced policymakers' confidence in the researcher's credibility.

For researchers, the barriers to enhancing motivation included the risk of sharing evidence that clashed with policymakers' expectations. This had the potential to create conflict between the two groups and limited the researcher's agency.

"*Sometimes, the evidence that we generate is at odds with policymaker position or expectations. That puts us in uncomfortable situations*." KII-R02, Researcher

To enhance the motivation of policymakers, researchers felt that sustaining engagement with policymakers would be important. Policymakers suggested that researchers should sensitise more policymakers on the importance of considering evidence during policymaking.

Capacity

Both groups felt that participation in KT activities enhanced the capacity of policymakers to use evidence. They felt that regular interaction with researchers and

For peer review only

evidence increased policymakers' ability to interpret and use evidence during policymaking:

"*The more they consume the evidence, the more they know how to consume the evidence.*" KII-R002, Researcher

A policymaker echoed this sentiment:

"*Okay, working with researchers much of now has built confidence in using information for making decisions, yes*." KII-PM07, Policymaker

A challenge identified by researchers in increasing the capacity of policymakers to appraise and use research evidence is the lack of time for policymakers to participate in capacity-building activities:

"*We know that policymakers do not have time*" KII-R02, Researcher

Policymakers also mentioned that short staffing in the department meant that they did not have the staffing available to continue training while attending to their everyday tasks.

Overall, both groups felt that more training and workshops to improve capacity to appraise and use evidence were needed when policymakers were available.

Relationships

Both groups reiterated that their relationships were valuable and had strengthened during the pandemic. For policymakers, an important facilitator for this was informal relationships with researchers. This meant that researchers were more accessible to them. Some noted that their relationship with some of the researchers was established even before joining the MoH, but further engagement deepened this relationship:

"*they weren't new to me, but of course the deepening of the relationships has come about now by us engaging further on this pandemic response that much I would say*" KII-PM01, Policymaker

According to the researchers, consistently sharing KT products that were timely and relevant made policymakers aware of the value of researchers which cultivated a good working relationship.

"*I think consistency is quite important. Ensuring the KT activities are regular, and the contact and the communication between policymakers and researchers is regular*" KII-R02, Researcher

Several barriers to relationship building were identified. From the researchers' perspective, these included 1) the constant personnel changes at the MoH; 2) being perceived as political players rather than evidence generators and 3) building and maintaining relationships took time and effort which was usually not accounted for as part of their research activities. From the policymakers' perspective, one barrier was the researchers' failure to meet all collaboration expectations. The policymaker expected more capacity building but conceded that due to COVID-19 restrictions, there were fewer training opportunities:

For peer review only

"..*one of our expectations for collaboration with KWTRP was knowledge transfer, and so that maybe was not achieved as yet. I'm sure there are still opportunities for us to go through that*." KII-PM05

Suggestions for addressing relationship barriers included strengthening institutional relationships and ensuring that engagement and communication between the two groups were sustained. Finally, researchers noted that they needed training on communicating with policymakers and navigating policy spaces without being seen as political.

Influence of KT activities on the COVID-19 response

Participating researchers felt that the KT activities they were involved in were useful in informing the COVID-19 response. This was reflected in the way policymakers regularly referred to briefs produced by the researchers and used evidence produced by them to inform their policymaking as stated by two researchers:

"I've seen action taken which I think is directly from the evidence that we've presented, and decisions being made based on some of the work that we've produced which I think is good to see" KII-R02, Researcher

"What I've seen is an action that was taken regarding a brief that we shared. I've seen in the media, action that has been taken and I've heard mentions of our work directly or indirectly by either the Director-General of Health, by the ministry of health" KII-R03, Researcher

According to the policymakers, the evidence disseminated by the researchers was used to inform COVID-19 policymaking as noted by several policymakers:

"*Much of the evidence shared was whether we should be able to strengthen some areas and also reinforce lockdowns. Some of the measures put in place have come from the reports that researchers provide*." KII-PM07, Policymaker

"*The briefs from genome sequencing have enabled us to map out hot spot areas which we used to give an advisory about movement. We also used the recommendations that came off the sero-surveillance evidence to advise the people in Nairobi on how to conduct themselves*". KII-PM09, Policymaker.

For peer review only

Table 3: Facilitators and barriers of evidence uptake

	Researchers		Policymakers	
	**Facilitators**	**Barriers**	**Facilitators**	**Barriers**
**Availability & accessibility**	• Institutional support and existing KT capacity<br>• Oral presentation of evidence	• Timeliness<br>• Limited availability of local data<br>• Lack of feedback mechanism	• Overall easy to read briefs<br>• Oral presentations<br>• Easy access to researchers	• Timeliness<br>• Unequal access to briefs<br>• Sometimes briefs were too technical or long
**Motivation**	• Producing relevant evidence to policymakers<br>• Availability of researchers to produce evidence	• Risk of sharing evidence that clashed with policymakers' expectations or views	• When evidence answered a priority question<br>• Availability and willingness of researchers to share evidence<br>• The positive feedback loop that was generated when evidence was used to inform prior decisions<br>• Credibility of researchers	• Sometimes researchers can be unresponsive/unavailable
**Capacity**	• Consistent interaction with policymakers and briefs increased capacity to understand and utilize evidence	• Policymakers lack time to take part in capacity development activities	• Regular interaction with research evidence & researchers	• Short staffing and increased responsibilities meant they did not have time for capacity building
**Relationships**	• Regular sharing of credible, timely and relevant evidence	• Constant personnel changes at the MoH<br>• Being viewed as political players<br>• Building and maintaining relationships was time-consuming	• Leveraging existing informal relationships with researchers	• When researchers failed to meet all of the policymakers' expectations for collaboration

Discussion

The KT activities described here promoted evidence uptake by increasing the accessibility and availability of evidence, motivating policymakers to use evidence, improving capacity, and enhancing the relationships between researchers and policymakers.

Some key facilitators of evidence uptake were highlighted. First were the tools used to communicate evidence which included rapid response evidence briefs and oral presentations. Evidence briefs describe an issue and provide evidence in a user-friendly, concise format. Similar to our finding, previous studies also report that the evidence briefs and summaries are well received by policymakers and increase the likelihood for policymakers to use them[11-13]. Second, strong relationships between researchers and policymakers were perceived as critical for KT activities as they enhanced evidence accessibility and built trust, similar to other studies[8,14,15]. Furthermore, policymakers in this study shared that informal relationships with researchers facilitated their professional relationships during the COVID-19 response. Likewise, Hyder et al. reported that when policymakers knew researchers socially, they were more open to considering their research[16]. However, while the value of informal relationships was noted, long-term engagement requires strengthening institutional relationships and formal networks especially considering the regular change in personnel at the MoH.

Lack of time has consistently been identified as a critical barrier to evidence uptake[8,14]. Often, the amount of time required by researchers to generate high-quality evidence does not match the policy makers' time frame for policymaking. In addition, researchers and policymakers have other responsibilities and find it challenging to dedicate time for KT activities. Finally, building relationships required continuous engagement, which was time intensive. Participants also reflected on the lack of skills to participate in KT and the insufficient institutional infrastructure to carry out KT. According to both groups, the main suggestion to address these barriers is to institutionalise KT and expand KT capacity at the research institution and MoH. Researchers with increased capacity and access to structured mechanisms for KT would be more motivated to take part in KT. Likewise, policymakers with increased capacity and institutional support can engage more effectively with research-type evidence. An effective way of strengthening the capacity of policymakers to use evidence is through training and mentorship programmes[17,18].

Policymakers noted that since COVID-19 was a crisis, they were more likely to seek and use COVID-19 related evidence. This largely explains the relative usefulness of our KT activities in informing the COVID-19 response. It was noted that institutions in LMICs are less prepared to conduct KT that responds to the pandemic[19]. In our case, we could leverage the EEVIDEM project that was already underway when the pandemic began, allowing us to respond to evidence demands readily. This further highlights the need to fully embed KT within the core functions of research and policymaking institutions. By doing this, researchers and policymakers are linked in a system that promotes a consistent exchange of knowledge and have the resources,

For peer review only

structures, and incentives to support KT initiatives that readily respond to public health emergencies.

Table 4: Suggestions to improve evidence uptake during public health emergencies using KT

Researchers should:	Policymakers should:
• Ensure KT products are relevant to policymakers' needs	• Institutionalize KT within policy-making spaces
• Improve readability of briefs by including tables and figures	• Make local evidence more available for researchers to better contextualize evidence
• Increase KT capacity in order to respond to evidence demands in a timely manner	• Actively engage with researchers to report on the usefulness of KT products and suggest areas of improvement
• Improve communication skills	• Strengthen and formalize institutional relationships with research institutions
• Sensitize more policymakers on the utility of research evidence	
• Conduct more trainings and workshop on KT for policymakers	
• Create open repositories for briefs	
• Maintain communication and interaction with policymakers	
• Institutionalize KT in research institutions	

Limitations

The researchers who carried out this study also participated in the KT activities described here, which presents a source of bias. The researchers recognised their positionality throughout the process and conducted reflective meetings to discuss how their biases may affect the study. The analysis was presented to KWTRP researchers for sense checking to see if their views were correctly represented. In addition, the study was presented to wider forums to check for objectivity and critique.

Conclusions

This study further highlights the value of KT in promoting evidence uptake during public health emergencies. It will be essential to build up from this experience and leverage the gains established during this time to institutionalise KT and sustain evidence use during policymaking going forward.

For peer review only - http://bmjopen.bmj.com/site/about/guidelines.xhtml

Contributors: FHG, AN, DK, EO, BT, ME, DK, and EB conceived the protocol; FHG and AN participated in the data collection and extraction; FHG and AN performed data analysis and FHG, JK, DK, EO, BT, ME, DK and EB participated in the article writing.

Funding: This manuscript is published with the permission of the Director of KEMRI and was funded by a Wellcome Trust Policy Engagement pilot award (215745); additional funds from a Wellcome Trust core grant awarded to the KEMRI-Wellcome Trust Research Programme (092654) supported this work. The funders had no role in study design, data analysis, decision to publish, drafting, or submission of the manuscript. The views expressed in the papers are for the authors and not for the organisations they represent.

Competing interests: None.

Patient consent for publication: Not required.

Data availability: No additional data available

References

1 Lancaster, K., Rhodes, T. & Rosengarten, M. Making evidence and policy in public health emergencies: Lessons from COVID-19 for adaptive evidence-making and intervention. *Evidence and Policy* **16**, 477-490 (2020).

2 Salajan, A., Tsolova, S., Ciotti, M. & Suk, J. E. To what extent does evidence support decision making during infectious disease outbreaks? A scoping literature review. *Evidence & Policy: A Journal of Research, Debate and Practice* **16**, 453-475, doi:10.1332/174426420X15808913064302 (2020).

3 El-Jardali, F., Bou-Karroum, L. & Fadlallah, R. Amplifying the role of knowledge translation platforms in the COVID-19 pandemic response. *Health Res Policy Syst* **18**, 58, doi:10.1186/s12961-020-00576-y (2020).

4 Straus, S. E., Tetroe, J. & Graham, I. Defining knowledge translation. *CMAJ : Canadian Medical Association journal = journal de l'Association medicale canadienne* **181**, 165-168, doi:10.1503/cmaj.081229 (2009).

5 Murunga, V. I., Oronje, R. N., Bates, I., Tagoe, N. & Pulford, J. Review of published evidence on knowledge translation capacity, practice and support among researchers and research institutions in low-and middle-income countries. *Health research policy and systems* **18**, 16 (2020).

6 Harvey, G., Marshall, R. J., Jordan, Z. & Kitson, A. L. Exploring the hidden barriers in knowledge translation: a case study within an academic community. *Qualitative health research* **25**, 1506-1517 (2015).

7 Kalbarczyk, A. *et al.* Barriers and facilitators to knowledge translation activities within academic institutions in low- and middle-income countries. *Health Policy and Planning*, doi:10.1093/heapol/czaa188 (2021).

8 Oliver, K., Innvar, S., Lorenc, T., Woodman, J. & Thomas, J. A systematic review of barriers to and facilitators of the use of evidence by policymakers. *BMC Health Serv Res* **14**, 2 (2014).

For peer review only

9 Tricco, A. C. *et al.* Barriers and facilitators to uptake of systematic reviews by policymakers and health care managers: a scoping review. *Implementation Science* **11**, 4, doi:10.1186/s13012-016-0370-1 (2016).
10 Foundation, H. Evidence-Informed Policymaking Strategy. (2018).
11 Moat, K. A., Lavis, J. N., Clancy, S. J., El-Jardali, F. & Pantoja, T. Evidence briefs and deliberative dialogues: perceptions and intentions to act on what was learnt. *Bulletin of the World Health Organization* **92**, 20-28 (2013).
12 Lavis, J. N., Permanand, G., Oxman, A. D., Lewin, S. & Fretheim, A. SUPPORT Tools for evidence-informed health Policymaking (STP) 13: Preparing and using policy briefs to support evidence-informed policymaking. *Health Research Policy and Systems* **7**, 1-9 (2009).
13 Dobbins, M., Jack, S., Thomas, H. & Kothari, A. Public health decision-makers' informational needs and preferences for receiving research evidence. *Worldviews Evid Based Nurs* **4**, 156-163, doi:10.1111/j.1741-6787.2007.00089.x (2007).
14 Innvaer, S., Vist, G., Trommald, M. & Oxman, A. Health policymakers' perceptions of their use of evidence: a systematic review. *Journal of health services research & policy* **7**, 239-244 (2002).
15 Jessani, N., Kennedy, C. & Bennett, S. Enhancing evidence-informed decision making: strategies for engagement between public health faculty and policymakers in Kenya. *Evid Policy* **13**, 225-253 (2016).
16 Hyder, A. A. *et al.* National policymakers speak out: are researchers giving them what they need? *Health Policy and Planning* **26**, 73-82, doi:10.1093/heapol/czq020 (2010).
17 Oronje, R. N., Murunga, V. I. & Zulu, E. M. Strengthening capacity to use research evidence in health sector policymaking: experience from Kenya and Malawi. *Health research policy and systems* **17**, 1-13 (2019).
18 Hawkes, S. *et al.* Strengthening capacity to apply health research evidence in policymaking: experience from four countries. *Health policy and planning* **31**, 161-170 (2016).
19 Mahendradhata, Y. & Kalbarczyk, A. Prioritizing knowledge translation in low- and middle-income countries to support pandemic response and preparedness. *Health Res Policy Syst* **19**, 5, doi:10.1186/s12961-020-00670-1 (2021).

Figures

Figure 1: Framework for assessing the usefulness of EEVIDEM activities

For peer review only

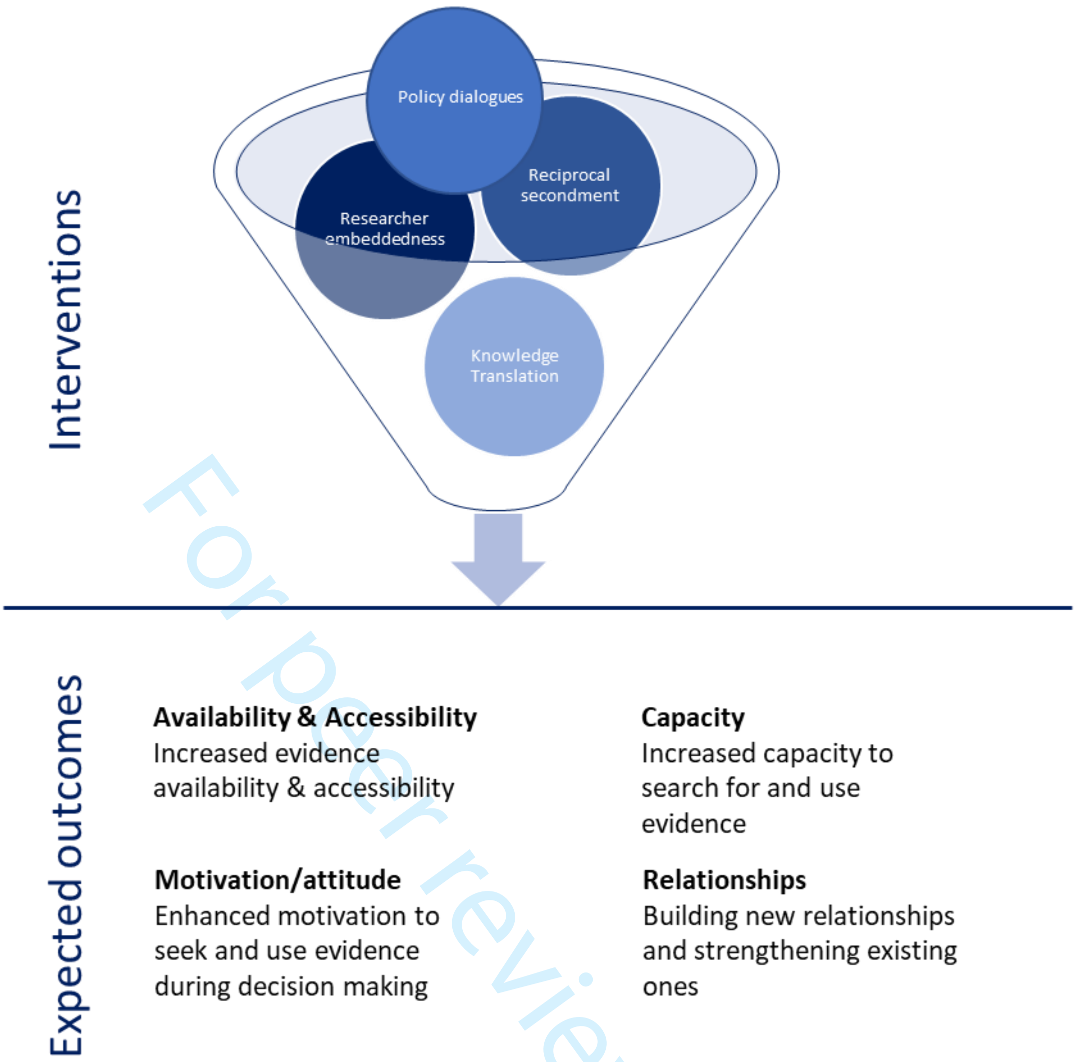

Figure 1: Framework for assessing the usefulness of EEVIDEM activities

COREQ (COnsolidated criteria for REporting Qualitative research) Checklist

A checklist of items that should be included in reports of qualitative research. You must report the page number in your manuscript where you consider each of the items listed in this checklist. If you have not included this information, either revise your manuscript accordingly before submitting or note N/A.

Topic	Item No.	Guide Questions/Description	Reported on Page No.
**Domain 1: Research team and reflexivity**			
Personal characteristics			
Interviewer/facilitator	1	Which author/s conducted the interview or focus group?	
Credentials	2	What were the researcher's credentials? E.g. PhD, MD	
Occupation	3	What was their occupation at the time of the study?	
Gender	4	Was the researcher male or female?	
Experience and training	5	What experience or training did the researcher have?	
Relationship with participants			
Relationship established	6	Was a relationship established prior to study commencement?	
Participant knowledge of the interviewer	7	What did the participants know about the researcher? e.g. personal goals, reasons for doing the research	
Interviewer characteristics	8	What characteristics were reported about the inter viewer/facilitator? e.g. Bias, assumptions, reasons and interests in the research topic	
**Domain 2: Study design**			
Theoretical framework			
Methodological orientation and Theory	9	What methodological orientation was stated to underpin the study? e.g. grounded theory, discourse analysis, ethnography, phenomenology, content analysis	
Participant selection			
Sampling	10	How were participants selected? e.g. purposive, convenience, consecutive, snowball	
Method of approach	11	How were participants approached? e.g. face-to-face, telephone, mail, email	
Sample size	12	How many participants were in the study?	
Non-participation	13	How many people refused to participate or dropped out? Reasons?	
Setting			
Setting of data collection	14	Where was the data collected? e.g. home, clinic, workplace	
Presence of non-participants	15	Was anyone else present besides the participants and researchers?	
Description of sample	16	What are the important characteristics of the sample? e.g. demographic data, date	
Data collection			
Interview guide	17	Were questions, prompts, guides provided by the authors? Was it pilot tested?	
Repeat interviews	18	Were repeat inter views carried out? If yes, how many?	
Audio/visual recording	19	Did the research use audio or visual recording to collect the data?	
Field notes	20	Were field notes made during and/or after the inter view or focus group?	
Duration	21	What was the duration of the inter views or focus group?	
Data saturation	22	Was data saturation discussed?	
Transcripts returned	23	Were transcripts returned to participants for comment and/or	

For peer review only

Topic	Item No.	Guide Questions/Description	Reported on Page No.
		correction?	
**Domain 3: analysis and findings**			
Data analysis			
Number of data coders	24	How many data coders coded the data?	
Description of the coding tree	25	Did authors provide a description of the coding tree?	
Derivation of themes	26	Were themes identified in advance or derived from the data?	
Software	27	What software, if applicable, was used to manage the data?	
Participant checking	28	Did participants provide feedback on the findings?	
Reporting			
Quotations presented	29	Were participant quotations presented to illustrate the themes/findings? Was each quotation identified? e.g. participant number	
Data and findings consistent	30	Was there consistency between the data presented and the findings?	
Clarity of major themes	31	Were major themes clearly presented in the findings?	
Clarity of minor themes	32	Is there a description of diverse cases or discussion of minor themes?	

Developed from: Tong A, Sainsbury P, Craig J. Consolidated criteria for reporting qualitative research (COREQ): a 32-item checklist for interviews and focus groups. *International Journal for Quality in Health Care*. 2007. Volume 19, Number 6: pp. 349 – 357

Once you have completed this checklist, please save a copy and upload it as part of your submission. DO NOT include this checklist as part of the main manuscript document. It must be uploaded as a separate file.

For peer review only

BMJ Open

Experience of Kenyan researchers and policy-makers with Knowledge translation during COVID-19: A qualitative interview study

Journal:	*BMJ Open*
Manuscript ID	bmjopen-2021-059501.R1
Article Type:	Original research
Date Submitted by the Author:	09-Mar-2022
Complete List of Authors:	Guleid, Fatuma; KEMRI-Wellcome Trust Research Programme Nairobi,<br>Njeru, Alex; KEMRI-Wellcome Trust Research Programme Nairobi<br>Kiptim, Joy; KEMRI-Wellcome Trust Research Programme Nairobi<br>Kamuya, Dorcas; KEMRI-Wellcome Trust Research Programme, Health Systems and Research Ethics; University of Oxford, Nuffield Department of medicine<br>Okiro, Emelda ; KEMRI-Wellcome Trust Research Programme Nairobi, Population Health ; University of Oxford, Nuffield department of medicine<br>Tsofa, Benjamin; KEMRI-Wellcome Trust Research Programme, Health Policy and Systems Research<br>English, Mike; KEMRI-Wellcome Trust Research Programme Nairobi, Health Services Unit; University of Oxford, Nuffield Department of Medicine<br>Molyneux, Sassy ; KEMRI-Wellcome Trust Research Programme, Health Systems and Research Ethics; University of Oxford, Nuffield Department of Medicine<br>Kariuki, David; Ministry of Health, Department of Health Policy and Research<br>Barasa, Edwine; KEMRI-Wellcome Trust Research Programme Nairobi, Health Economics Research Unit; University of Oxford, Nuffield Department of Medicine
**Primary Subject Heading**:	Health policy
Secondary Subject Heading:	Evidence based practice, Global health
Keywords:	COVID-19, Health policy < HEALTH SERVICES ADMINISTRATION & MANAGEMENT, Public health < INFECTIOUS DISEASES

SCHOLARONE™
Manuscripts

I, the Submitting Author has the right to grant and does grant on behalf of all authors of the Work (as defined in the below author licence), an exclusive licence and/or a non-exclusive licence for contributions from authors who are: i) UK Crown employees; ii) where BMJ has agreed a CC-BY licence shall apply, and/or iii) in accordance with the terms applicable for US Federal Government officers or employees acting as part of their official duties; on a worldwide, perpetual, irrevocable, royalty-free basis to BMJ Publishing Group Ltd ("BMJ") its licensees and where the relevant Journal is co-owned by BMJ to the co-owners of the Journal, to publish the Work in this journal and any other BMJ products and to exploit all rights, as set out in our licence.

The Submitting Author accepts and understands that any supply made under these terms is made by BMJ to the Submitting Author unless you are acting as an employee on behalf of your employer or a postgraduate student of an affiliated institution which is paying any applicable article publishing charge ("APC") for Open Access articles. Where the Submitting Author wishes to make the Work available on an Open Access basis (and intends to pay the relevant APC), the terms of reuse of such Open Access shall be governed by a Creative Commons licence – details of these licences and which Creative Commons licence will apply to this Work are set out in our licence referred to above.

Other than as permitted in any relevant BMJ Author's Self Archiving Policies, I confirm this Work has not been accepted for publication elsewhere, is not being considered for publication elsewhere and does not duplicate material already published. I confirm all authors consent to publication of this Work and authorise the granting of this licence.

For peer review only

Experience of Kenyan researchers and policy-makers with Knowledge translation during COVID-19: A qualitative interview study

Fatuma H Guleid[1], Alex Njeru[1], Joy Kiptim[1], Dorcas Kamuya[2,7], Emelda Okiro[3,7], Benjamin Tsofa[2], Mike English[4,7], Sassy Molyneux[2,7], David Kariuki[6], Edwine Barasa[5,7]

1. Policy Engagement & Knowledge Translation Unit, KEMRI-Wellcome Trust Research Programme, Kenya
2. Health Systems & Research Ethics Department, KEMRI-Wellcome Trust Research Programme, Kenya
3. Population Health Unit, KEMRI-Wellcome Trust Research Programme, Kenya
4. Health Services Unit, KEMRI-Wellcome Trust Research Programme, Kenya
5. Health Economics Research Unit, KEMRI-Wellcome Trust Research Programme, Kenya
6. Department of Health Policy and Research, Ministry of Health, Kenya
7. Center for Tropical Medicine and Global Health, Nuffield Department of Medicine, University of Oxford, Oxford, UK

Correspondence to Fatuma H Guleid: fguleid@kemri-wellcome.org

Abstract

Objective Researchers at the KEMRI-Wellcome Trust Research Programme (KWTRP) carried out Knowledge Translation (KT) activities to support policy-makers as the Kenyan Government responded to the COVID-19 pandemic. We assessed the usefulness of these activities to identify the facilitators and barriers to KT and suggest actions that facilitate KT in similar settings.

Design The study adopted a qualitative interview study design.

Settings and participants Researchers at KWTRP in Kenya who were involved in KT activities during the COVID-19 pandemic (n=6) were selected to participate in key informant interviews to describe their experience. In addition, the policy-makers with whom these researchers engaged were invited to participate (n=11). Data was collected from March 2021-August 2021.

Analysis A thematic analysis approach was adopted using a predetermined framework to develop a coding structure consisting of the core thematic areas. Any other theme that emerged in the coding process was included.

Results Both groups reported that the KT activities increased evidence availability and accessibility, enhanced policy-makers' motivation to use evidence, improved capacity to use research evidence, and strengthened relationships. Policy-makers shared that a key facilitator of this was the knowledge products shared and the regular interaction with researchers. Both groups mentioned a key barrier was the timeliness of generating evidence, which was exacerbated by the pandemic. Both groups felt it was important to institutionalise KT to improve readiness to respond to public health emergencies.

For peer review only

Conclusion This study provides a real-world example of the use of KT during a public health crisis. It further highlights the need to institutionalise KT in research and policy institutions in African countries to respond readily to public health emergencies.

Strength and limitations of this study

- This study employed in-depth interviews to elucidate the engagement between researchers and policy-makers and how evidence is utilised in a pandemic.
- Including researchers as participants in the study may have introduced bias.

Introduction

The COVID-19 pandemic has required policy-makers to make decisions that have significant health, social and economic consequences. Incorporating the best available research evidence to guide decision-making is essential for an effective response. However, public health emergencies (PHEs) present several challenges to evidence-informed decision-making. These include: (i) a rapidly increasing and evolving evidence base; (ii) increased uncertainty and risk; (iii) and sheer time pressure to make quick decisions[1]. Knowledge translation (KT) is best placed to respond to this challenge[2,3]. The World Health Organisation defines knowledge translation as "the synthesis, exchange, and application of knowledge by relevant stakeholders to accelerate the benefits of global and local innovation in strengthening health systems and improving people's health". KT models and frameworks have been successfully used to bridge the gap between evidence and policy. There are four key models of KT[4]: (i) push models where researchers disseminate research evidence to those who can use it; (ii) pull models where research users ask for evidence; (iii) the exchange models which depend on partnerships between researchers and research users for mutual benefit; and (iv) integrated model where linkages and exchange occurs across institutions and systems.

There are several documented barriers to effective KT for researchers and policy-makers in low-middle income regions such as Africa. For researchers, these include lack of capacity to plan and implement KT, poor relationships between researchers and policy-makers, competing demands for time, a lack of institutional incentives to participate in KT, the complexity of the policy process, and difficulty in communicating research evidence[5-7]. For policy-makers, the most frequent barriers include lack of accessible evidence, lack of timely research output, and poor capacity to appraise and understand research evidence[8]. These barriers are exacerbated in the context of PHEs. While there have been several studies on KT in Africa, the literature on the use of KT in responding to PHEs in Africa is limited. More research is needed to inform how institutions in Africa can conduct KT effectively in emergency settings.

Enhancing Evidence-Informed Decision Making for health (EEVIDEM)

The EEVIDEM project was a KEMRI-Wellcome Trust Research Programme (KWTRP) initiative based on the KT push and exchange models that sought to promote evidence-informed decision-making in Kenya using four interventions. The first intervention was researcher embeddedness into policy-making spaces like technical working groups. The second intervention was reciprocal secondment. For example, a policy-maker from the Ministry of Health (MoH) spends 50% of their working hours at

For peer review only - http://bmjopen.bmj.com/site/about/guidelines.xhtml

KWTRP, allowing them to participate in research activities. Likewise, one researcher from KWTRP spends 50% of their time at the MoH. The third intervention was hosting regular policy dialogues to provide a platform for stakeholders to reflect critically on policy issues. The fourth intervention was knowledge synthesis and dissemination (hereafter collectively referred to as "KT activities"). This intervention involved generating evidence and developing and disseminating KT products to policy-makers and is the paper's focus.

While KWTRP had engaged in KT activities before EEVIDEM, these were ad hoc, inconsistent, and driven by few individual researcher initiatives. Institutionalising KT means taking steps to ensure that the systems, structures, resources and support needed for KT to become standard practices are available. The EEVIDEM project supported the institutionalisation of KT within KWTRP by providing financial resources for KT, staffing with KT capacities, facilitating linkages between researchers and policy-makers, providing technical support to researchers to develop and implement KT strategies, and coordinating KT in the institution.

We sought to describe the experiences of the researchers and policy-makers involved in KT activities that support the COVID-19 response and assess the influence of these activities. The lessons learned from these experiences can strengthen the processes that promote evidence-informed responses during PHEs in African settings.

Methods

Study setting

In Kenya, the Ministry of Health (MoH) has established organisational structures to support using evidence in policy-making. The Kenya Medical Research Institute (KEMRI), of which KWTRP is a part, is mandated to generate this evidence. During the pandemic, the MoH established the National COVID-19 taskforce. Its membership is drawn from the MoH and other government agencies, research and academic institutions, United Nations agencies, civil society organisations, and the private sector. The taskforce coordinates and mobilises technical advice to the MoH and advises other ministries on appropriate measures. The taskforce's action points are implemented within the MoH's departmental structure, which includes the Director General's Office and the Emergency Operations Centre (EOC). KWTRP researchers provided research evidence and engaged with policy-makers within these structures.

EEVIDEM Conceptual framework

To guide the assessment of the influence of KWTRP's KT activities on evidence uptake during the COVID-19 pandemic, we adapted a determinant framework (Fig. 1) that conceptualises determinants that have been found to influence KT outcomes[8-10]. This framework emphasises four overlapping and interacting determinants, namely: (i) availability and accessibility of evidence; (ii) attitude/motivation to search for and use evidence; (iii) capacity to appraise and utilise evidence; and (iv) the relationship between the researchers and policy-makers.

KT activities

For peer review only

Initially, the researchers synthesised evidence on questions they thought were of high priority. As the pandemic progressed, policy-makers started engaging with researchers to identify priority questions and co-produce COVID-19 evidence. The evidence was packaged as rapid response evidence briefs and/evidence summaries. Dissemination was primarily through email, and in some cases, researchers were invited to do oral presentations.

Study design

This study adopted a qualitative interview cross-sectional design. A total of 17 key informant interviews (KIIs) were carried out on two groups: researchers and policy-makers (Table 1). All KWTRP researchers involved in KT activities to support the COVID-19 response were interviewed (n=6). The selected researchers identified the policy-makers they engaged during their KT activities who were then invited to participate in the study. We identified 21 eligible policy-makers. Out of this, 11 policy-makers were interviewed, after which a point of information saturation was reached. EEVIDEM's conceptual framework was used to develop the interview guides and facilitate the interviews. Separate guides were used for researchers and policy-makers. Two researchers (FG and AN) conducted all the interviews virtually using Microsoft Teams after each participant provided informed consent. Each interview lasted between 30-60 minutes and was audio-recorded using an encrypted audio recorder, except for one policy-maker who declined consent to audio recording - the interview was captured by a note-taker in handwritten notes. Data was collected from March to August 2021.

Patients and public involvement

No patients were involved in this study.

Table 1: Institutions and departments of participants in the study

Researchers	Policy-makers
Health Economics Research Unit	Ministry of Health
Health Systems and Policy Research group	Ministry of Health-Emergency Operations Center (EOC)
Health Services Research unit	Ministry of Health
Virus Epidemiology and Control Research group	Presidential Policy and Strategy Unit
Virus Epidemiology and Control Research group	Ministry of Health-EOC
Policy Engagement and Knowledge Translation	World Health Organisation-Kenya office

For peer review only

	Ministry of Health-EOC
	Ministry of Health-Office of the Cabinet Administrative Secretary of Health
	Ministry of Health-Office of the Director-General of Health
	Ministry of Health-Department of Health Policy & Research
	Kilifi County Department of Health Services

Data management and analysis

Audio recordings were transcribed verbatim in MS Word. The transcripts were coded using NVIVO qualitative data analysis software (QSR International Pty Ltd. Version 12, 2014). For analysis, a predetermined coding structure consisting of the core thematic areas of the conceptual framework was used. Any other theme that emerged in the coding process was included. Code reports were generated and analysed once all data were coded in their respective themes (FG & AN).

Ethical considerations

This study received ethical approval from KEMRI's Scientific and Ethics Review Committee. Information sheets detailing the study description were shared with all participants before the interviews and signed consent was obtained.

Results

Fifty-Six rapid response evidence briefs and summaries were developed. These briefs shared evidence from primary studies, including modelling studies, genomic surveillance of SARS-CoV-2, seroprevalence of SARS-CoV-2 antibodies, health systems assessment and economics studies, clinical surveillance, and from the synthesis of existing evidence on topics requested by policy-makers (Table 2). Briefs were 2-4 pages long and included key messages, brief background, and evidence findings.

Table 2: Summary of briefs

Topic	Number
Seroprevalence and genomic surveillance of SARS-CoV-2	21
Other COVID-19 related briefs	26
Clinical surveillance	9

For peer review only

Participants described several facilitators and barriers that shaped their experience, as summarised in Table 3.

1. **Accessibility and availability**

The researchers felt that their KT efforts increased evidence availability and accessibility. A significant facilitator of this was the support of existing KT capacity in KWTRP through EEVIDEM that provided guidance and supported researchers in the consistent and timely development of KT products.

"*it has been the existence of the structures that we've managed to create with this platform [EEVIDEM]. It's something that we don't emphasise enough, having that pool of people*." KII-R05, Researcher

Policy-makers also reported that the briefs enhanced their access to research evidence. They highlighted the format of the briefs as important in making evidence more accessible as the briefs presented evidence in concise and simplified language.

"*I found the format fairly useful. I like that there was a section summarising what the rest of the brief held. It was beneficial as one can quickly garner some of the key evidence from that particular brief very quickly*" KII-PM08, Policy-maker

Both groups reported the importance of augmenting written briefs with an oral presentation of evidence which provided them with an opportunity to discuss the evidence and reduce misinterpretation:

"*They [the researchers] present most of the information and not only presented in writing, they normally present there [at the MoH]. So you can always seek clarification should there be a need to.*" KII-PM01, Policy-maker

"*The presentations are best because they involve you interacting with the people who are making the decisions or who are going to interpret the evidence. So, you get to communicate far more information unlike these other modes of communication*" KII-03, Researcher

The main barrier to evidence availability and accessibility for both groups was timeliness. The time it took to generate and synthesise high-quality evidence was often longer than the time within which evidence was needed for timely policy-making.

"*Sometimes they are required urgently, and I have to turn them around while ensuring that they're of reasonable quality which can be a challenge*" KII-R05, Researcher

"*The challenge that is experienced is the length of time from once a request is sent out for a certain matter to the time a brief is submitted on the same*" KII-PM01, Policy-maker

Other barriers mentioned by researchers included the limited availability of local data, making it difficult for researchers to adapt evidence to local settings. Another barrier was the lack of a feedback mechanism. Some researchers were unaware of how useful briefs were to policy-makers as they rarely got direct feedback. Policy-makers

For peer review only

mentioned two more barriers. One was the length of the briefs; they felt that when a brief was long or too technical, it lowered the accessibility of the evidence. In addition, policy-makers shared that there was unequal access to evidence amongst them:

"*In my position, getting evidence is not as difficult; however, other officers within the directorate who find it a bit difficult to access the evidence and are told write officially or show that it comes through like DGs office or PS office so that they can access the evidence.*" KII-PM01, Policy-maker

Researchers highlighted three factors that could improve evidence availability and accessibility. These included expanding KT capacity in KWTRP, increasing availability of local data to researchers to support the contextualisation of evidence to the local setting, and more feedback from policy-makers to help researchers determine if policy-makers found evidence easier to use through their KT activities. Suggestions by policy-makers included making briefs even easier to read by using more illustrations and tables and depositing briefs on open platforms so that more policy-makers have access to evidence.

Motivation/attitude

Both groups reported that regular sharing of briefs enhanced policy-maker motivation to seek and use research evidence. A key facilitator was the relevance of the evidence. Motivation was enhanced as long as the evidence answered a priority question:

"*To a certain extent, the larger the crisis, the more the need for research. However, the endless loop for motivation would be the need to answer a question at the forefront of policy-makers' minds*" KII-PM03, Policy-maker

Both groups also felt that the availability and willingness of researchers to generate and share evidence with policy-makers was an important motivator.

"*I think just knowing that we are available to them is a huge motivator. That someone else can search for evidence and summarise it and then present it to them is a huge motivator.*" KII-R06, Researcher

Policy-makers mentioned other facilitators including the positive feedback loop generated when evidence-informed policies were implemented and prior engagement between the two groups. These factors enhanced policy-makers' confidence in the researcher's credibility.

For researchers, the barriers to enhancing motivation included the risk of sharing evidence that clashed with policy-makers' expectations. This could create conflict between the two groups and limit the researcher's agency.

"*Sometimes, the evidence that we generate is at odds with policy-makers' position or expectations. That puts us in uncomfortable situations.*" KII-R02, Researcher

To enhance the motivation of policy-makers, researchers felt that sustaining engagement with policy-makers would be necessary. Policy-makers suggested that researchers should sensitise more policy-makers on the importance of considering evidence during policy-making.

For peer review only - http://bmjopen.bmj.com/site/about/guidelines.xhtml

Capacity

Both groups felt that participation in KT activities enhanced the capacity of policy-makers to use evidence. They felt that regular interaction with researchers and evidence increased policy-makers' ability to interpret and use evidence during policy-making:

"*Working with researchers has built confidence in using information for making decisions, yes*." KII-PM07, Policy-maker

A challenge identified by researchers in increasing the capacity of policy-makers to appraise and use research evidence is the lack of time for policy-makers to participate in capacity-building activities:

"*We know that policy-makers do not have time*" KII-R02, Researcher

Policy-makers also mentioned that short staffing in the department meant that they did not have the staffing available to continue training while attending to their everyday tasks.

Overall, both groups felt that more KT training and workshops to improve capacity to appraise and use evidence were needed when policy-makers were available.

Relationships

Both groups reiterated that their relationships were valuable and had strengthened during the pandemic. For policy-makers, an important facilitator for this was informal relationships with researchers. This meant that researchers were more accessible to them. Some noted that their relationship with some of the researchers was established even before joining the MoH, but further engagement deepened this relationship:

"*they weren't new to me, but of course, the deepening of the relationships has come about now by us engaging further on this pandemic response that much I would say*" KII-PM01, Policy-maker

According to the researchers, consistently sharing KT products that were timely and relevant made policy-makers aware of researchers' value, which cultivated a good working relationship. However, several barriers to relationship building were identified. From the researchers' perspective, these include: (i) the constant personnel changes at the MoH; (ii) being perceived as political players rather than evidence generators; and (iii) building and maintaining relationships took time and effort which was usually not accounted for as part of their research activities. From the policy-makers' perspective, one barrier was the researchers' failure to meet all collaboration expectations. The policy-maker expected more capacity building but conceded that due to COVID-19 restrictions, there were fewer opportunities. Suggestions for addressing relationship barriers included strengthening institutional relationships and ensuring that engagement and communication between the two groups were sustained. Finally, researchers noted that they needed training on communicating with policy-makers and navigating policy spaces without being seen as political.

Influence of KT activities on the COVID-19 response

For peer review only - http://bmjopen.bmj.com/site/about/guidelines.xhtml

Participating researchers felt that the KT activities they were involved in were useful in informing the COVID-19 response. This was reflected in the way policy-makers regularly referred to briefs produced by the researchers and used evidence produced. According to the policy-makers, the evidence disseminated by the researchers was used to inform COVID-19 policy-making as noted by several policy-makers:

"*Much of the evidence shared was whether we should be able to strengthen some areas and also reinforce lockdowns. Some of the measures put in place have come from the reports that researchers provide*." KII-PM07, Policy-maker

"*The briefs from genome sequencing have enabled us to map out hot spot areas which we used to give an advisory about movement. We also used the recommendations that came off the sero-surveillance evidence to advise the people in Nairobi on how to conduct themselves*". KII-PM09, Policy-maker.

For peer review only

Table 3: Facilitators and barriers of evidence uptake

	Researchers		Policy-makers	
	**Facilitators**	**Barriers**	**Facilitators**	**Barriers**
**Availability & accessibility**	• Institutional support and existing KT capacity<br>• Oral presentation of evidence	• Timeliness<br>• Limited availability of local data<br>• Lack of feedback mechanism	• Overall easy to read briefs<br>• Oral presentations<br>• Easy access to researchers	• Timeliness<br>• Unequal access to briefs<br>• Sometimes briefs were too technical or long
**Motivation**	• Producing relevant evidence to policy-makers<br>• Availability of researchers to produce evidence	• Risk of sharing evidence that clashed with policy-makers' expectations or views	• When evidence answered a priority question<br>• Availability of researchers to share evidence<br>• The positive feedback loop that was generated when evidence was used to inform prior decisions<br>• Credibility of researchers	• Sometimes researchers can be unresponsive/unavailable
**Capacity**	• Consistent interaction with policy-makers and briefs increased capacity to understand and utilise evidence	• Policy-makers lack time to take part in capacity development activities	• Regular interaction with research evidence & researchers	• Short staffing and increased responsibilities meant they did not have time for capacity building
**Relationships**	• Regular sharing of credible, timely and relevant evidence	• Constant personnel changes at the MoH<br>• Being viewed as political players<br>• Building and maintaining relationships was time-consuming	• Leveraging existing informal relationships with researchers	• When researchers failed to meet all of the policy-makers' expectations for collaboration

Discussion

The KT activities described here promoted evidence use for COVID-19 response. Some key facilitators of evidence uptake were highlighted. First were the tools used to communicate evidence, including rapid response evidence briefs and oral presentations. Similar to our finding, previous studies also report that the evidence briefs and summaries are well received by policy-makers and increase the likelihood of using them[11-13]. Second, strong relationships between researchers and policy-makers were perceived as critical for KT activities as they enhanced evidence accessibility and built trust, similar to other studies[8,14,15]. Furthermore, policy-makers in this study shared that informal relationships with researchers facilitated their professional relationships during the COVID-19 response. Likewise, Hyder et al. reported that when policy-makers knew researchers socially, they were more open to considering their research[16].

Lack of time has consistently been identified as a critical barrier to evidence uptake[8,14]. Often, the amount of time required by researchers to generate high-quality evidence does not match the policy makers' time frame for policy-making. In addition, researchers and policy-makers have other responsibilities and find it challenging to dedicate time to KT activities. Finally, building relationships required continuous engagement, which was time-intensive. Participants also reflected on the lack of skills to participate in KT and the insufficient institutional infrastructure to carry out KT. According to both groups, the main suggestion to address these barriers is institutionalising KT and expanding KT capacity at the research institution and MoH.

It was noted that institutions in LMICs are less prepared to conduct KT that responds to the pandemic[17]. The experiences and outcomes we report highlight the urgent need to institutionalise KT within research and government institutions in Africa to respond to PHEs adequately. For example, through institutionalisation, funds for KT can be planned for as part of an institution's budget. Such investments would strengthen KT capacity amongst researchers and policy-makers. Researchers with increased capacity and access to structured mechanisms for KT would be more motivated to take part in KT. Likewise, policy-makers with increased capacity and institutional support can engage more effectively with research-type evidence. Institutionalisation can also overcome the problems with timeliness in terms of low staffing. Institutions can hire more KT experts to provide on-demand evidence promptly. This would put less pressure on researchers who balance their daily research activities while responding to new evidence demands. Institutionalisation also promotes relationship building and partnerships, especially with the regular staff turnover at Ministries of Health. Such formal partnerships build trust and credibility and facilitate mutual understanding, which is critical when responding to crises. In our case, we could leverage on the EEVIDEM project that was already underway when the pandemic began. EEVIDEM provided a system that promoted a consistent exchange of knowledge and provided the resources, structures, and incentives to support KT.

Institutionalisation of KT is particularly needed in African countries, where the use of evidence during decision making is still challenged by insufficient KT capacity and weak mechanisms to support evidence-informed decision-making[17]. Research

For peer review only

institutions in Africa are significantly contributing to COVID-19 research[18-20]. However, to ensure that this research is used to inform local responses, evidence use and KT should be institutionalised.

Table 4: Suggestions to improve evidence uptake during PHEs using KT

Researchers should:	Policy-makers should:
• Ensure KT products are relevant to policy-makers' needs	• Institutionalise KT within policy-making spaces
• Improve readability of briefs by including tables and figures	• Make local evidence more available for researchers to contextualise evidence better
• Increase KT capacity to respond to evidence demands promptly	• Actively engage with researchers to report on the usefulness of KT products and suggest areas of improvement
• Improve communication skills	• Strengthen and formalise institutional relationships with research institutions
• Sensitise more policy-makers on the utility of research evidence	
• Conduct more training and workshop on KT for policy-makers	
• Create open repositories for briefs	
• Maintain communication and interaction with policy-makers	
• Institutionalise KT in research institutions	

Limitations

The researchers in this study also participated in the KT activities described here which may impact analysis. However, the participatory nature of our KT approach makes it inherently collaborative which inadvertently sets the researcher up as a participant. To address trustworthiness, the researchers were transparent about their relationship to the topic of the investigation and maintained reflexivity throughout the process. In addition, the analysis was presented to the participants for sense checking to see if their views were correctly represented. The study was also presented to broader forums to check for objectivity. The study interviewed 17 participants which limited statistical generalisability. However, as is consistent with qualitative studies, the aim was not statistical generalisability, but rather analytical generalizability, typically achieved with relatively small sample sizes that facilitate in-depth exploration

of participants' perspectives[21]. Therefore, the analytical themes elucidated by the study are relevant to contexts similar to the study context.

Conclusions

The key learning from this study is the importance of institutionalising KT in research and government institutions to support PHEs. In this study, we sought to increase evidence uptake by enhancing four main determinants of uptake: accessibility, motivation, capacity, and relationships. We found that by using this framework, we had relative success in informing policy-making during COVID-19. The novelty of the approach lies in the context within which it is applied. By accurately recording our experiences, interventions and outcomes we can derive lessons to inform the establishment of institutionalised KT that responds to PHEs (Table 4).

Contributors: FHG, AN, DK, EO, BT, ME, DK, and EB conceived the protocol; FHG and AN participated in the data collection and extraction; FHG and AN performed data analysis and FHG, JK, DK, EO, BT, ME, DK and EB participated in the article writing.

Funding: This manuscript is published with the permission of the Director of KEMRI and was funded by a Wellcome Trust award (215745); additional funds from a Wellcome Trust core grant awarded to KWTRP (092654) supported this work. The funders had no role in study design, data analysis, decision to publish, drafting, or submission of the manuscript. The views expressed in the papers are for the authors and not for the organisations they represent.

Competing interests: None.

Patient consent for publication: Not required.

For peer review only

References

1 Salajan, A., Tsolova, S., Ciotti, M. & Suk, J. E. To what extent does evidence support decision making during infectious disease outbreaks? A scoping literature review. *Evidence & Policy: A Journal of Research, Debate and Practice* **16**, 453-475, doi:10.1332/174426420X15808913064302 (2020).

2 El-Jardali, F., Bou-Karroum, L. & Fadlallah, R. Amplifying the role of knowledge translation platforms in the COVID-19 pandemic response. *Health Res Policy Syst* **18**, 58, doi:10.1186/s12961-020-00576-y (2020).

3 Lancaster, K., Rhodes, T. & Rosengarten, M. Making evidence and policy in public health emergencies: Lessons from COVID-19 for adaptive evidence-making and intervention. *Evidence and Policy* **16**, 477-490 (2020).

4 Oborn, E., Barrett, M. & Racko, G. *Knowledge translation in healthcare: A review of the literature*. (Citeseer, 2010).

5 Murunga, V. I., Oronje, R. N., Bates, I., Tagoe, N. & Pulford, J. Review of published evidence on knowledge translation capacity, practice and support among researchers and research institutions in low-and middle-income countries. *Health research policy and systems* **18**, 16 (2020).

6 Harvey, G., Marshall, R. J., Jordan, Z. & Kitson, A. L. Exploring the hidden barriers in knowledge translation: a case study within an academic community. *Qualitative health research* **25**, 1506-1517 (2015).

7 Kalbarczyk, A. *et al.* Barriers and facilitators to knowledge translation activities within academic institutions in low- and middle-income countries. *Health Policy and Planning*, doi:10.1093/heapol/czaa188 (2021).

8 Oliver, K., Innvar, S., Lorenc, T., Woodman, J. & Thomas, J. A systematic review of barriers to and facilitators of the use of evidence by policymakers. *BMC Health Serv Res* **14**, 2 (2014).

9 Tricco, A. C. *et al.* Barriers and facilitators to uptake of systematic reviews by policy makers and health care managers: a scoping review. *Implementation Science* **11**, 4, doi:10.1186/s13012-016-0370-1 (2016).

10 Foundation, H. Evidence-Informed Policymaking Strategy. (2018).

11 Moat, K. A., Lavis, J. N., Clancy, S. J., El-Jardali, F. & Pantoja, T. Evidence briefs and deliberative dialogues: perceptions and intentions to act on what was learnt. *Bulletin of the World Health Organization* **92**, 20-28 (2013).

12 Lavis, J. N., Permanand, G., Oxman, A. D., Lewin, S. & Fretheim, A. SUPPORT Tools for evidence-informed health Policymaking (STP) 13: Preparing and using policy briefs to support evidence-informed policymaking. *Health Research Policy and Systems* **7**, 1-9 (2009).

13 Dobbins, M., Jack, S., Thomas, H. & Kothari, A. Public health decision-makers' informational needs and preferences for receiving research evidence. *Worldviews Evid Based Nurs* **4**, 156-163, doi:10.1111/j.1741-6787.2007.00089.x (2007).

14 Innvaer, S., Vist, G., Trommald, M. & Oxman, A. Health policy-makers' perceptions of their use of evidence: a systematic review. *Journal of health services research & policy* **7**, 239-244 (2002).

15 Jessani, N., Kennedy, C. & Bennett, S. Enhancing evidence-informed decision making: strategies for engagement between public health faculty and policymakers in Kenya. *Evid Policy* **13**, 225-253 (2016).

16 Hyder, A. A. *et al.* National policy-makers speak out: are researchers giving them what they need? *Health Policy and Planning* **26**, 73-82, doi:10.1093/heapol/czq020 (2010).

17 Mahendradhata, Y. & Kalbarczyk, A. Prioritizing knowledge translation in low- and middle-income countries to support pandemic response and preparedness. *Health Res Policy Syst* **19**, 5, doi:10.1186/s12961-020-00670-1 (2021).

For peer review only

18 Mbogning Fonkou, M. D. *et al.* COVID-19 Pandemic Related Research in Africa: Bibliometric Analysis of Scholarly Output, Collaborations and Scientific Leadership. *Int J Environ Res Public Health* **18**, doi:10.3390/ijerph18147273 (2021).

19 Kana, M. A., LaPorte, R. & Jaye, A. Africa's contribution to the science of the COVID-19/SARS-CoV-2 pandemic. *BMJ Global Health* **6**, e004059, doi:10.1136/bmjgh-2020-004059 (2021).

20 Guleid, F. H. *et al.* A bibliometric analysis of COVID-19 research in Africa. *BMJ Glob Health* **6**, doi:10.1136/bmjgh-2021-005690 (2021).

21 Polit, D. F. & Beck, C. T. Generalization in quantitative and qualitative research: Myths and strategies. *International journal of nursing studies* **47**, 1451-1458 (2010).

Figures

Figure 1: Framework for assessing the usefulness of EEVIDEM activities

For peer review only

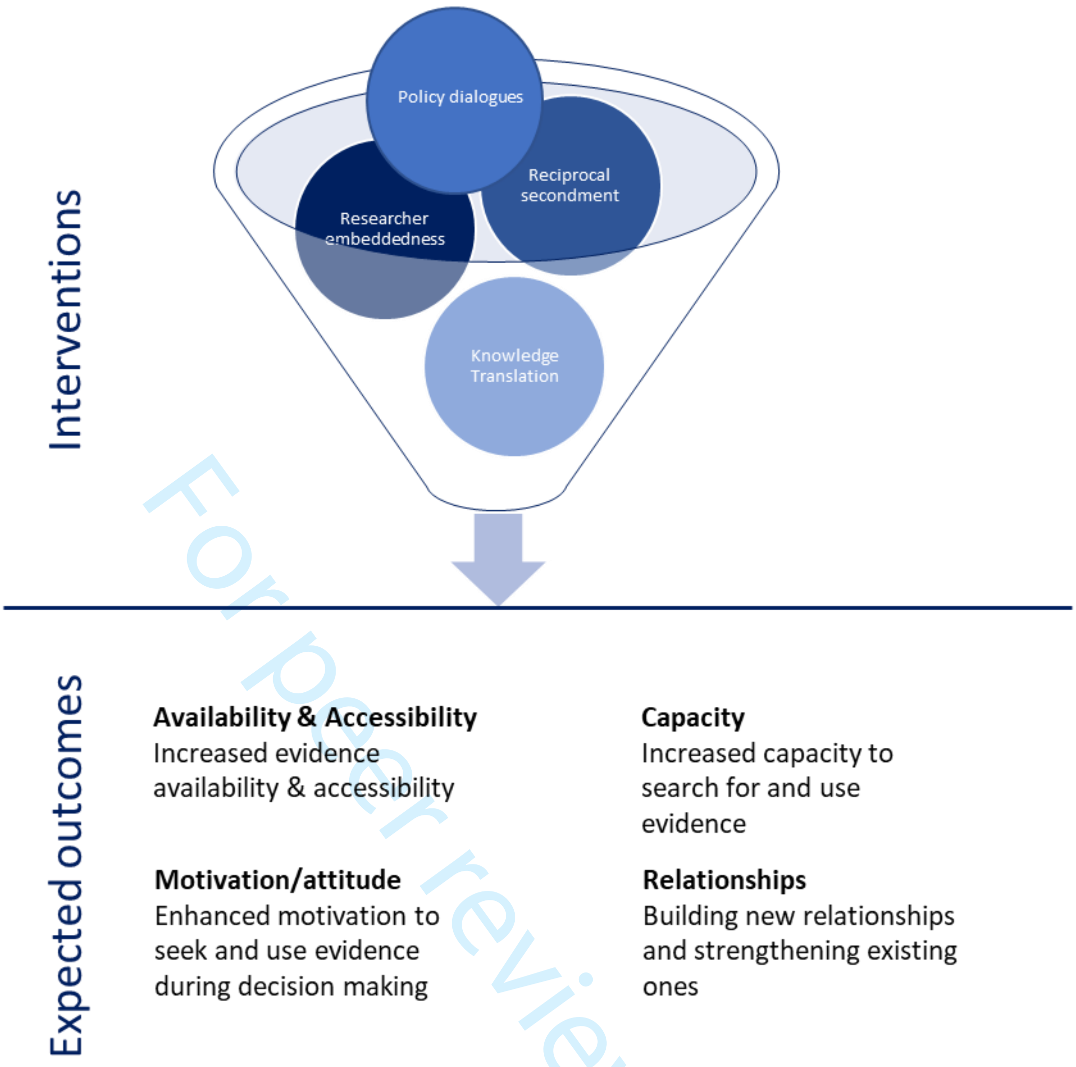

Figure 1: Framework for assessing the usefulness of EEVIDEM activities

For peer review only

COREQ (COnsolidated criteria for REporting Qualitative research) Checklist

A checklist of items that should be included in reports of qualitative research. You must report the page number in your manuscript where you consider each of the items listed in this checklist. If you have not included this information, either revise your manuscript accordingly before submitting or note N/A.

Topic	Item No.	Guide Questions/Description	Reported on Page No.
**Domain 1: Research team and reflexivity**			
Personal characteristics			
Interviewer/facilitator	1	Which author/s conducted the interview or focus group?	
Credentials	2	What were the researcher's credentials? E.g. PhD, MD	
Occupation	3	What was their occupation at the time of the study?	
Gender	4	Was the researcher male or female?	
Experience and training	5	What experience or training did the researcher have?	
Relationship with participants			
Relationship established	6	Was a relationship established prior to study commencement?	
Participant knowledge of the interviewer	7	What did the participants know about the researcher? e.g. personal goals, reasons for doing the research	
Interviewer characteristics	8	What characteristics were reported about the inter viewer/facilitator? e.g. Bias, assumptions, reasons and interests in the research topic	
**Domain 2: Study design**			
Theoretical framework			
Methodological orientation and Theory	9	What methodological orientation was stated to underpin the study? e.g. grounded theory, discourse analysis, ethnography, phenomenology, content analysis	
Participant selection			
Sampling	10	How were participants selected? e.g. purposive, convenience, consecutive, snowball	
Method of approach	11	How were participants approached? e.g. face-to-face, telephone, mail, email	
Sample size	12	How many participants were in the study?	
Non-participation	13	How many people refused to participate or dropped out? Reasons?	
Setting			
Setting of data collection	14	Where was the data collected? e.g. home, clinic, workplace	
Presence of non-participants	15	Was anyone else present besides the participants and researchers?	
Description of sample	16	What are the important characteristics of the sample? e.g. demographic data, date	
Data collection			
Interview guide	17	Were questions, prompts, guides provided by the authors? Was it pilot tested?	
Repeat interviews	18	Were repeat inter views carried out? If yes, how many?	
Audio/visual recording	19	Did the research use audio or visual recording to collect the data?	
Field notes	20	Were field notes made during and/or after the inter view or focus group?	
Duration	21	What was the duration of the inter views or focus group?	
Data saturation	22	Was data saturation discussed?	
Transcripts returned	23	Were transcripts returned to participants for comment and/or	

For peer review only

Topic	Item No.	Guide Questions/Description	Reported on Page No.
		correction?	
**Domain 3: analysis and findings**			
Data analysis			
Number of data coders	24	How many data coders coded the data?	
Description of the coding tree	25	Did authors provide a description of the coding tree?	
Derivation of themes	26	Were themes identified in advance or derived from the data?	
Software	27	What software, if applicable, was used to manage the data?	
Participant checking	28	Did participants provide feedback on the findings?	
Reporting			
Quotations presented	29	Were participant quotations presented to illustrate the themes/findings? Was each quotation identified? e.g. participant number	
Data and findings consistent	30	Was there consistency between the data presented and the findings?	
Clarity of major themes	31	Were major themes clearly presented in the findings?	
Clarity of minor themes	32	Is there a description of diverse cases or discussion of minor themes?	

Developed from: Tong A, Sainsbury P, Craig J. Consolidated criteria for reporting qualitative research (COREQ): a 32-item checklist for interviews and focus groups. *International Journal for Quality in Health Care*. 2007. Volume 19, Number 6: pp. 349 – 357

Once you have completed this checklist, please save a copy and upload it as part of your submission. DO NOT include this checklist as part of the main manuscript document. It must be uploaded as a separate file.

For peer review only

BMJ Open

Experience of Kenyan researchers and policy-makers with knowledge translation during COVID-19: a qualitative interview study

Journal:	*BMJ Open*
Manuscript ID	bmjopen-2021-059501.R2
Article Type:	Original research
Date Submitted by the Author:	07-Apr-2022
Complete List of Authors:	Guleid, Fatuma; KEMRI-Wellcome Trust Research Programme Nairobi,<br>Njeru, Alex; KEMRI-Wellcome Trust Research Programme Nairobi<br>Kiptim, Joy; KEMRI-Wellcome Trust Research Programme Nairobi<br>Kamuya, Dorcas; KEMRI-Wellcome Trust Research Programme, Health Systems and Research Ethics; University of Oxford, Nuffield Department of medicine<br>Okiro, Emelda ; KEMRI-Wellcome Trust Research Programme Nairobi, Population Health ; University of Oxford, Nuffield department of medicine<br>Tsofa, Benjamin; KEMRI-Wellcome Trust Research Programme, Health Policy and Systems Research<br>English, Mike; KEMRI-Wellcome Trust Research Programme Nairobi, Health Services Unit; University of Oxford, Nuffield Department of Medicine<br>Molyneux, Sassy ; KEMRI-Wellcome Trust Research Programme, Health Systems and Research Ethics; University of Oxford, Nuffield Department of Medicine<br>Kariuki, David; Ministry of Health, Department of Health Policy and Research<br>Barasa, Edwine; KEMRI-Wellcome Trust Research Programme Nairobi, Health Economics Research Unit; University of Oxford, Nuffield Department of Medicine
**Primary Subject Heading**:	Health policy
Secondary Subject Heading:	Evidence based practice, Global health
Keywords:	COVID-19, Health policy < HEALTH SERVICES ADMINISTRATION & MANAGEMENT, Public health < INFECTIOUS DISEASES

SCHOLARONE™
Manuscripts

I, the Submitting Author has the right to grant and does grant on behalf of all authors of the Work (as defined in the below author licence), an exclusive licence and/or a non-exclusive licence for contributions from authors who are: i) UK Crown employees; ii) where BMJ has agreed a CC-BY licence shall apply, and/or iii) in accordance with the terms applicable for US Federal Government officers or employees acting as part of their official duties; on a worldwide, perpetual, irrevocable, royalty-free basis to BMJ Publishing Group Ltd ("BMJ") its licensees and where the relevant Journal is co-owned by BMJ to the co-owners of the Journal, to publish the Work in this journal and any other BMJ products and to exploit all rights, as set out in our licence.

The Submitting Author accepts and understands that any supply made under these terms is made by BMJ to the Submitting Author unless you are acting as an employee on behalf of your employer or a postgraduate student of an affiliated institution which is paying any applicable article publishing charge ("APC") for Open Access articles. Where the Submitting Author wishes to make the Work available on an Open Access basis (and intends to pay the relevant APC), the terms of reuse of such Open Access shall be governed by a Creative Commons licence – details of these licences and which Creative Commons licence will apply to this Work are set out in our licence referred to above.

Other than as permitted in any relevant BMJ Author's Self Archiving Policies, I confirm this Work has not been accepted for publication elsewhere, is not being considered for publication elsewhere and does not duplicate material already published. I confirm all authors consent to publication of this Work and authorise the granting of this licence.

For peer review only

Experience of Kenyan researchers and policy-makers with knowledge translation during COVID-19: a qualitative interview study

Fatuma H Guleid[1], Alex Njeru[1], Joy Kiptim[1], Dorcas Kamuya[2,7], Emelda Okiro[3,7], Benjamin Tsofa[2], Mike English[4,7], Sassy Molyneux[2,7], David Kariuki[6], Edwine Barasa[5,7]

1. Policy Engagement & Knowledge Translation Unit, KEMRI-Wellcome Trust Research Programme, Kenya
2. Health Systems & Research Ethics Department, KEMRI-Wellcome Trust Research Programme, Kenya
3. Population Health Unit, KEMRI-Wellcome Trust Research Programme, Kenya
4. Health Services Unit, KEMRI-Wellcome Trust Research Programme, Kenya
5. Health Economics Research Unit, KEMRI-Wellcome Trust Research Programme, Kenya
6. Department of Health Policy and Research, Ministry of Health, Kenya
7. Center for Tropical Medicine and Global Health, Nuffield Department of Medicine, University of Oxford, Oxford, UK

Correspondence to:

Fatuma H Guleid
fguleid@kemri-wellcome.org

For peer review only

Abstract

Objectives Researchers at the KEMRI-Wellcome Trust Research Programme (KWTRP) carried out Knowledge Translation (KT) activities to support policy-makers as the Kenyan Government responded to the COVID-19 pandemic. We assessed the usefulness of these activities to identify the facilitators and barriers to KT and suggest actions that facilitate KT in similar settings.

Design The study adopted a qualitative interview study design.

Setting and participants Researchers at KWTRP in Kenya who were involved in KT activities during the COVID-19 pandemic (n=6) were selected to participate in key informant interviews to describe their experience. In addition, the policy-makers with whom these researchers engaged were invited to participate (n=11). Data were collected from March 2021-August 2021.

Analysis A thematic analysis approach was adopted using a predetermined framework to develop a coding structure consisting of the core thematic areas. Any other theme that emerged in the coding process was included.

Results Both groups reported that the KT activities increased evidence availability and accessibility, enhanced policy-makers' motivation to use evidence, improved capacity to use research evidence, and strengthened relationships. Policy-makers shared that a key facilitator of this was the knowledge products shared and the regular interaction with researchers. Both groups mentioned that a key barrier was the timeliness of generating evidence, which was exacerbated by the pandemic. They felt it was

important to institutionalise KT to improve readiness to respond to public health emergencies.

Conclusion This study provides a real-world example of the use of KT during a public health crisis. It further highlights the need to institutionalise KT in research and policy institutions in African countries to respond readily to public health emergencies.

Strength and limitations of this study

- This study employed in-depth interviews to elucidate the engagement between researchers and policy-makers and how evidence is utilised in a pandemic.
- The study provides lessons that can inform KT practice in similar settings
- Including researchers as participants in the study may have introduced bias.

For peer review only

Introduction

The COVID-19 pandemic has required policy-makers to make decisions that have significant health, social and economic consequences. Incorporating the best available research evidence to guide decision-making is essential for an effective response. However, public health emergencies (PHEs) present several challenges to evidence-informed decision-making (EIDM). These include: (i) a rapidly increasing and evolving evidence base; (ii) increased uncertainty and risk; (iii) and sheer time pressure to make quick decisions[1]. Knowledge translation (KT) is best placed to respond to this challenge[2,3]. The World Health Organisation defines knowledge translation as "the synthesis, exchange, and application of knowledge by relevant stakeholders to accelerate the benefits of global and local innovation in strengthening health systems and improving people's health". There are four key models of KT[4]: (i) push models where researchers disseminate research evidence to those who can use it; (ii) pull models where research users ask for evidence; (iii) the exchange models which depend on partnerships between researchers and research users for mutual benefit; and (iv) integrated models where linkages and exchange occurs across institutions and systems.

There are several documented barriers to effective KT in low-middle income regions such as Africa. For researchers, these include lack of capacity to plan and implement KT, poor relationships between researchers and policy-makers, competing demands for time, a lack of institutional incentives to participate in KT, the complexity of the policy process, and difficulty in communicating research evidence[5-7]. For policy-makers, the most frequent barriers include lack of accessible evidence, lack of timely research output, and poor capacity to appraise and understand research evidence[8]. These barriers are exacerbated in the context of PHEs. While there have been several studies on KT in Africa, the literature on the use of KT in responding to PHEs in Africa is limited. More research is needed to inform how institutions in Africa can conduct KT effectively in emergency settings.

Enhancing Evidence-Informed Decision Making for health (EEVIDEM)

The EEVIDEM project was a KEMRI-Wellcome Trust Research Programme (KWTRP) initiative based on the KT push, exchange, and integrated models that sought to promote EIDM in Kenya using four interventions. The first intervention was researcher embeddedness into policy-making spaces like technical working groups. The second intervention was reciprocal secondment. In this arrangement, a policy-maker from the Ministry of Health (MoH) spends 50% of their working hours at KWTRP, allowing them to participate in research activities. Likewise, one researcher from KWTRP spends 50% of their time at the MoH. The third intervention was hosting regular policy dialogues. The fourth intervention was knowledge synthesis and dissemination (hereafter collectively referred to as "KT activities"). This intervention involved generating evidence and disseminating KT products to policy-makers and is the paper's focus.

While KWTRP had engaged in KT activities before EEVIDEM, these were ad hoc, inconsistent, and driven by few individual researcher initiatives. Institutionalising KT means taking steps to ensure that the systems, structures, resources and support needed for KT to become standard practices are available. The EEVIDEM project supported the institutionalisation of KT within KWTRP by facilitating the development of a policy engagement and KT strategy, creating mechanisms for documentation and evaluation of KT efforts, providing financial resources for KT, staffing with KT capacities, formation of structures that support linkages between researchers and policy-makers, and providing technical support to researchers to develop and implement KT strategies.

We sought to describe the experiences of the researchers and policy-makers involved in EEVIDEM's KT activities that supported the COVID-19 response and assess the influence of these activities. The lessons learned from these experiences can strengthen the processes that promote evidence-informed responses during PHEs in African settings.

Methods

Study setting

In Kenya, the Ministry of Health (MoH) has established organisational structures to support using evidence in policy-making. The Kenya Medical Research Institute (KEMRI), of which KWTRP is a part, is mandated to generate this evidence. During the pandemic, the MoH established the National COVID-19 taskforce. Its membership was drawn from the MoH and other government agencies, research and academic institutions, United Nations agencies, civil society organisations, and the private sector. The taskforce coordinates and mobilises technical advice to the MoH and advises other ministries on appropriate measures. The taskforce's action points are implemented within the MoH's departmental structure, which includes the Director General's Office and the Emergency Operations Centre (EOC). KWTRP researchers provided research evidence and engaged with policy-makers within these structures.

EEVIDEM conceptual framework

To guide the assessment of the influence of KWTRP's KT activities on evidence uptake during the COVID-19 pandemic, we adapted a determinant framework (Fig. 1)

For peer review only

that conceptualises determinants that have been found to influence KT outcomes[8-10]. This framework emphasises four overlapping and interacting determinants, namely: (i) availability and accessibility of evidence; (ii) attitude/motivation to search for and use evidence; (iii) capacity to appraise and utilise evidence; and (iv) the relationship between the researchers and policy-makers.

KT activities

Initially, the researchers synthesised evidence on questions they thought were of high priority. As the pandemic progressed, policy-makers started engaging with researchers to identify priority questions and co-produce COVID-19 evidence. The evidence was packaged as rapid response evidence briefs and/evidence summaries. Dissemination was primarily through email, and in some cases, researchers were invited to do oral presentations.

Study design

This study adopted a qualitative interview cross-sectional design. A total of 17 key informant interviews (KIIs) were carried out on two groups: researchers and policy-makers (Table 1). All KWTRP researchers involved in KT activities (n=6) to support the COVID-19 response were interviewed until data saturation was achieved. The selected researchers identified the policy-makers they engaged during their KT activities who were then invited to participate in the study. We identified 21 eligible policy-makers. Out of this, 11 policy-makers were interviewed, after which a point of information saturation was reached. EEVIDEM's conceptual framework was used to develop the interview guides and facilitate the interviews. Separate guides were used for researchers and policy-makers. Two researchers (FG and AN) conducted all the interviews virtually using Microsoft Teams after each participant provided informed consent. Each interview lasted between 30-60 minutes and was audio-recorded using an encrypted audio recorder, except for one policy-maker who declined consent to audio recording - the interview was captured by a note-taker in handwritten notes. Data were collected from March to August 2021.

Patients and public involvement

There was no patient or public involvement in the study.

Table 1: Institutions and departments of participants in the study

Researchers	Policy-makers
Health Economics Research Unit	Ministry of Health
Health Systems and Policy Research group	Ministry of Health-Emergency Operations Center (EOC)
Health Services Research unit	Ministry of Health

For peer review only

Virus Epidemiology and Control Research group	Presidential Policy and Strategy Unit
Virus Epidemiology and Control Research group	Ministry of Health-EOC
Policy Engagement and Knowledge Translation	World Health Organisation-Kenya office
	Ministry of Health-EOC
	Ministry of Health-Office of the Cabinet Administrative Secretary of Health
	Ministry of Health-Office of the Director-General of Health
	Ministry of Health-Department of Health Policy & Research
	Kilifi County Department of Health Services

Data management and analysis

Audio recordings were transcribed verbatim in MS Word. The transcripts were coded using NVIVO qualitative data analysis software (QSR International Pty Ltd. Version 12, 2014). For analysis, a predetermined coding structure consisting of the core thematic areas of the conceptual framework was used. Any other theme that emerged in the coding process was included. Code reports were generated and analysed once all data were coded in their respective themes (FG & AN).

Ethical considerations

This study received ethical approval from KEMRI's Scientific and Ethics Review Committee. Information sheets detailing the study description were shared with all participants before the interviews and signed consent was obtained.

Results

56 rapid response evidence briefs and summaries were developed. These briefs shared evidence from primary studies, including modelling studies, genomic surveillance of SARS-CoV-2, seroprevalence of SARS-CoV-2 antibodies, health systems assessment and economics studies, clinical surveillance, and the synthesis of existing evidence on topics requested by policy-makers (Table 2). Briefs were 2-4 pages long and included key messages, brief background, and evidence findings.

Table 2: Summary of briefs

Topic	Number

For peer review only - http://bmjopen.bmj.com/site/about/guidelines.xhtml

Seroprevalence and genomic surveillance of SARS-CoV-2	21
Other COVID-19 related briefs	26
Clinical surveillance	9

Participants described several facilitators and barriers that shaped their experience, as summarised in Table 3.

Accessibility and availability

The researchers felt that their KT efforts increased evidence availability and accessibility. A significant facilitator of this was the support of existing KT capacity in KWTRP through EEVIDEM that provided guidance and supported researchers in the consistent and timely development of KT products.

"*it has been the existence of the structures that we've managed to create with this platform [EEVIDEM]. It's something that we don't emphasise enough, having that pool of people*." KII-R05, Researcher

Policy-makers also reported that the briefs enhanced their access to research evidence. They highlighted the format of the briefs as important in making evidence more accessible as they were concise and used simple language.

"*I found the format fairly useful. I like that there was a section summarising what the rest of the brief held. It was beneficial as one can quickly garner some of the key evidence from that particular brief very quickly*" KII-PM08, Policy-maker

Both groups reported the importance of augmenting written briefs with an oral presentation of evidence which provided them with an opportunity to discuss the evidence and reduce misinterpretation:

"*They [the researchers] present most of the information and not only presented in writing, they normally present there [at the MoH]. So you can always seek clarification should there be a need to.*" KII-PM01, Policy-maker

"*The presentations are best because they involve you interacting with the people who are making the decisions or who will interpret the evidence. So, you get to communicate far more information unlike these other modes of communication"* KII-03, Researcher

The main barrier to evidence availability and accessibility for both groups was timeliness. The time it took to generate and synthesise high-quality evidence was often longer than the time within which evidence was needed for timely policy-making.

"*Sometimes they are required urgently, and I have to turn them around while ensuring that they're of reasonable quality which can be a challenge*" KII-R05, Researcher

For peer review only

"*The challenge that is experienced is the length of time from once a request is sent out for a certain matter to the time a brief is submitted on the same*" KII-PM01, Policy-maker

Other barriers mentioned by researchers included the limited availability of local data, making it difficult for researchers to adapt evidence to local settings. In addition, the lack of a feedback mechanism left some researchers unaware of how useful briefs were to policy-makers as they rarely got direct feedback. Policy-makers mentioned two more barriers. One was the length of the briefs; they felt that when a brief was long or too technical, it lowered the accessibility of the evidence. In addition, policy-makers shared that there was unequal access to evidence amongst them:

"*In my position, getting evidence is not as difficult; however, other officers within the directorate find it a bit difficult to access the evidence and are told write officially or show that it comes through like DGs office or PS office so that they can access the evidence.*" KII-PM01, Policy-maker

Researchers highlighted three factors that could improve evidence availability and accessibility. These included expanding KT capacity in KWTRP, increasing availability of local data to researchers to support the contextualisation of evidence to the local setting, and more feedback from policy-makers to help researchers determine if policy-makers found evidence easier to use through their KT activities. Suggestions by policy-makers included making briefs even easier to read by using more illustrations and tables and depositing briefs on open platforms so that more policy-makers have access to evidence.

Motivation/attitude

Both groups reported that regular sharing of briefs enhanced policy-maker motivation to seek and use research evidence. A key facilitator was the relevance of the evidence. Motivation was enhanced as long as the evidence answered a priority question:

"*To a certain extent, the larger the crisis, the more the need for research. However, the endless loop for motivation would be the need to answer a question at the forefront of policy-makers' minds*" KII-PM03, Policy-maker

Both groups also felt that the availability and willingness of researchers to generate and share evidence with policy-makers was an important motivator.

"*I think just knowing that we are available to them is a huge motivator. That someone else can search for evidence and summarise it and then present it to them is a huge motivator*." KII-R06, Researcher

Policy-makers mentioned other facilitators including the positive feedback loop generated when evidence-informed policies were implemented and prior engagement between the two groups. These factors enhanced policy-makers' confidence in the researcher's credibility.

For researchers, the barriers to enhancing motivation included the risk of sharing evidence that clashed with policy-makers' expectations. This could create conflict between the two groups and limit the researcher's agency.

For peer review only

"*Sometimes, the evidence we generate is at odds with policy-makers' position or expectations. That puts us in uncomfortable situations*." KII-R02, Researcher

To enhance the motivation of policy-makers, researchers felt that sustaining engagement with policy-makers would be necessary. Policy-makers suggested that researchers should sensitise more policy-makers on the importance of considering evidence during policy-making.

Capacity

Both groups felt that participation in KT activities enhanced the capacity of policy-makers to use evidence. They felt that regular interaction with researchers and evidence increased policy-makers' ability to interpret and use evidence during policy-making:

"*Working with researchers has built confidence in using information for making decisions, yes*." KII-PM07, Policy-maker

A challenge identified by researchers in increasing the capacity of policy-makers to appraise and use research evidence is the lack of time for policy-makers to participate in capacity-building activities:

"*We know that policy-makers do not have time*" KII-R02, Researcher

Policy-makers also mentioned that short staffing in the department meant that they did not have the staffing available to continue training while attending to their everyday tasks.

Overall, both groups felt that more KT training and workshops to improve capacity to appraise and use evidence were needed when policy-makers were available.

Relationships

Both groups reiterated that their relationships were valuable and had strengthened during the pandemic. For policy-makers, an important facilitator for this was informal relationships with researchers. This meant that researchers were more accessible to them. Some noted that their relationship with some of the researchers was established even before joining the MoH, but further engagement deepened this relationship:

"*they weren't new to me, but of course, the deepening of the relationships has come about now by us engaging further on this pandemic response that much I would say*" KII-PM01, Policy-maker

According to the researchers, consistently sharing KT products that were timely and relevant made policy-makers aware of researchers' value, which cultivated a good working relationship. However, several barriers to relationship building were identified. From the researchers' perspective, these include: (i) the constant personnel changes at the MoH; (ii) being perceived as political players rather than evidence generators; and (iii) building and maintaining relationships took time and effort which was usually not accounted for as part of their research activities. From the policy-makers' perspective, one barrier was the researchers' failure to meet all collaboration expectations. The policy-maker expected more capacity building but conceded that

For peer review only

due to COVID-19 restrictions, there were fewer opportunities. Suggestions for addressing relationship barriers included strengthening institutional relationships and ensuring that engagement and communication between the two groups were sustained. Finally, researchers noted that they needed training on communicating with policy-makers and navigating policy spaces without being seen as political.

Influence of KT activities on the COVID-19 response

Participating researchers felt that the KT activities they were involved in were useful in informing the COVID-19 response. This was reflected in the way policy-makers regularly referred to briefs produced by the researchers and used evidence produced. According to the policy-makers, the evidence disseminated by the researchers was used to inform COVID-19 policy-making as noted by several policy-makers:

"*Much of the evidence shared was whether we should be able to strengthen some areas and also reinforce lockdowns. Some of the measures put in place have come from the reports that researchers provide*." KII-PM07, Policy-maker

"*The briefs from genome sequencing have enabled us to map out hot spot areas which we used to give an advisory about movement. We also used the recommendations that came off the sero-surveillance evidence to advise the people in Nairobi on how to conduct themselves*". KII-PM09, Policy-maker.

For peer review only

Table 3: Facilitators and barriers of evidence uptake

	Researchers		Policy-makers	
	**Facilitators**	**Barriers**	**Facilitators**	**Barriers**
**Availability & accessibility**	• Institutional support and existing KT capacity<br>• Oral presentation of evidence	• Timeliness<br>• Limited availability of local data<br>• Lack of feedback mechanism	• Overall easy to read briefs<br>• Oral presentations<br>• Easy access to researchers	• Timeliness<br>• Unequal access to briefs<br>• Sometimes briefs were too technical or long
**Motivation**	• Producing relevant evidence to policy-makers<br>• Availability of researchers to produce evidence	• Risk of sharing evidence that clashed with policy-makers' expectations or views	• When evidence answered a priority question<br>• Availability of researchers to share evidence<br>• The positive feedback loop that was generated when evidence was used to inform prior decisions<br>• Credibility of researchers	• Sometimes researchers can be unresponsive/unavailable
**Capacity**	• Consistent interaction with policy-makers and briefs increased capacity to understand and utilise evidence	• Policy-makers lack time to take part in capacity development activities	• Regular interaction with research evidence & researchers	• Short staffing and increased responsibilities meant they did not have time for capacity building
**Relationships**	• Regular sharing of credible, timely and relevant evidence	• Constant personnel changes at the MoH<br>• Being viewed as political players<br>• Building and maintaining relationships was time-consuming	• Leveraging existing informal relationships with researchers	• When researchers failed to meet all of the policy-makers' expectations for collaboration

Discussion

The KT activities described here promoted evidence use for COVID-19 response. Some key facilitators of evidence uptake were highlighted. First were the tools used to communicate evidence, including rapid response evidence briefs and oral presentations. Similar to our finding, previous studies also report that the evidence briefs and summaries are well received by policy-makers and increase the likelihood of using them[11-13]. Second, strong relationships between researchers and policy-makers were perceived as critical for KT activities as they enhanced evidence accessibility and built trust, similar to other studies[8,14,15]. Furthermore, policy-makers in this study shared that informal relationships with researchers facilitated their professional relationships during the COVID-19 response. Likewise, Hyder et al. reported that when policy-makers knew researchers socially, they were more open to considering their research[16].

Lack of time has consistently been identified as a critical barrier to evidence uptake[8,14]. Often, the amount of time required by researchers to generate high-quality evidence does not match the policy makers' time frame for policy-making. In addition, researchers and policy-makers have other responsibilities and find it challenging to dedicate time to KT activities. Finally, building relationships required continuous engagement, which was time-intensive. Participants also reflected on the lack of skills to participate in KT and the insufficient institutional infrastructure to carry out KT. According to both groups, the main suggestion to address these barriers is institutionalising KT and expanding KT capacity at the research institution and MoH.

It was noted that institutions in LMICs are less prepared to conduct KT that responds to the pandemic[17]. The experiences and outcomes we report highlight the urgent need to institutionalise KT within research and government institutions in Africa to respond to PHEs adequately. For example, through institutionalisation, funds for KT can be planned for as part of an institution's budget. Such investments would strengthen KT capacity in both research and policy institutions. Researchers with increased capacity and access to structured mechanisms for KT would be more motivated to take part in KT. Likewise, policy-makers with increased capacity and institutional support can engage more effectively with research-type evidence. Institutionalisation can also overcome the problems with timeliness in terms of low staffing. Institutions can hire more KT experts to provide on-demand evidence promptly. This would put less pressure on researchers who balance their daily research activities while responding to new evidence demands. Institutionalisation also promotes relationship building and partnerships, especially with the regular staff turnover at MoHs. Such formal partnerships build trust and credibility and facilitate mutual understanding, which is critical when responding to crises. In our case, we leveraged on the EEVIDEM project that was already underway when the pandemic began. EEVIDEM provided a system that promoted a consistent exchange of knowledge and provided the resources, structures, and incentives to support KT.

Institutionalisation of KT is particularly needed in African countries, where the use of evidence during decision making is still challenging [17]. Research institutions in Africa are significantly contributing to COVID-19 research[18-20]. However, to ensure that this

For peer review only

research is used to inform local responses, evidence use and KT should be institutionalised.

Table 4: Suggestions to improve evidence uptake during PHEs using KT

Researchers should:	Policy-makers should:
• Ensure KT products are relevant to policy-makers' needs	• Institutionalise KT within policy-making spaces
• Improve readability of briefs by including tables and figures	• Make local evidence more available for researchers to contextualise evidence better
• Increase KT capacity to respond to evidence demands promptly	• Actively engage with researchers to report on the usefulness of KT products and suggest areas of improvement
• Improve communication skills	• Strengthen and formalise institutional relationships with research institutions
• Sensitise more policy-makers on the utility of research evidence	
• Conduct more training and workshop on KT for policy-makers	
• Create open repositories for briefs	
• Maintain communication and interaction with policy-makers	
• Institutionalise KT in research institutions	

Limitations

The researchers in this study also participated in the KT activities described here which may impact analysis. However, the participatory nature of our KT approach made it inherently collaborative which inadvertently set the researcher up as a participant. To address trustworthiness, the researchers were transparent about their relationship to the topic of the investigation and maintained reflexivity throughout the process. In addition, the analysis was presented to the participants for sense checking to see if their views were correctly represented. The study was also presented to broader forums to check for objectivity. Finally, we interviewed 17 participants which limited statistical generalisability. However, as is consistent with qualitative studies, the aim was not statistical generalisability, but rather analytical generalizability, typically achieved with relatively small sample sizes that facilitate in-depth exploration

of participants' perspectives[21]. Therefore, the analytical themes elucidated by the study are relevant to contexts similar to the study context.

Conclusions

The key learning from this study is the importance of institutionalising KT in research and government institutions to support PHEs. In this study, we sought to increase evidence uptake by enhancing four main determinants of uptake: accessibility, motivation, capacity, and relationships. We found that by using this framework, we had relative success in informing policy-making during COVID-19. The novelty of the approach lies in the context within which it is applied. By accurately recording our experiences, interventions, and outcomes we can derive lessons to inform the establishment of institutionalised KT that responds to PHEs (Table 4).

Contributors: FHG, AN, DK, EO, BT, ME, SM, DK, and EB conceived the protocol; FHG and AN participated in the data collection and extraction; FHG and AN performed data analysis and FHG, JK, DK, EO, BT, ME, SM, DK and EB participated in the article writing.

Funding: This manuscript is published with the permission of the Director of KEMRI and was funded by a Wellcome Trust award (215745); additional funds from a Wellcome Trust core grant awarded to KWTRP (092654) supported this work. The funders had no role in study design, data analysis, decision to publish, drafting, or submission of the manuscript. The views expressed in the papers are for the authors and not for the organisations they represent.

Competing interests: None.

Patient consent for publication: Not required.

Data availability statement: All data relevant to the study are included in the article or uploaded as supplementary information

For peer review only

References

1 Salajan, A., Tsolova, S., Ciotti, M. & Suk, J. E. To what extent does evidence support decision making during infectious disease outbreaks? A scoping literature review. *Evidence & Policy: A Journal of Research, Debate and Practice* **16**, 453-475, doi:10.1332/174426420X15808913064302 (2020).

2 El-Jardali, F., Bou-Karroum, L. & Fadlallah, R. Amplifying the role of knowledge translation platforms in the COVID-19 pandemic response. *Health Res Policy Syst* **18**, 58, doi:10.1186/s12961-020-00576-y (2020).

3 Lancaster, K., Rhodes, T. & Rosengarten, M. Making evidence and policy in public health emergencies: Lessons from COVID-19 for adaptive evidence-making and intervention. *Evidence and Policy* **16**, 477-490 (2020).

4 Oborn, E., Barrett, M. & Racko, G. *Knowledge translation in healthcare: A review of the literature*. (Citeseer, 2010).

5 Murunga, V. I., Oronje, R. N., Bates, I., Tagoe, N. & Pulford, J. Review of published evidence on knowledge translation capacity, practice and support among researchers and research institutions in low-and middle-income countries. *Health research policy and systems* **18**, 16 (2020).

6 Harvey, G., Marshall, R. J., Jordan, Z. & Kitson, A. L. Exploring the hidden barriers in knowledge translation: a case study within an academic community. *Qualitative health research* **25**, 1506-1517 (2015).

7 Kalbarczyk, A. *et al.* Barriers and facilitators to knowledge translation activities within academic institutions in low- and middle-income countries. *Health Policy and Planning*, doi:10.1093/heapol/czaa188 (2021).

8 Oliver, K., Innvar, S., Lorenc, T., Woodman, J. & Thomas, J. A systematic review of barriers to and facilitators of the use of evidence by policymakers. *BMC Health Serv Res* **14**, 2 (2014).

9 Tricco, A. C. *et al.* Barriers and facilitators to uptake of systematic reviews by policy makers and health care managers: a scoping review. *Implementation Science* **11**, 4, doi:10.1186/s13012-016-0370-1 (2016).

10 Foundation, H. Evidence-Informed Policymaking Strategy. (2018).

11 Moat, K. A., Lavis, J. N., Clancy, S. J., El-Jardali, F. & Pantoja, T. Evidence briefs and deliberative dialogues: perceptions and intentions to act on what was learnt. *Bulletin of the World Health Organization* **92**, 20-28 (2013).

12 Lavis, J. N., Permanand, G., Oxman, A. D., Lewin, S. & Fretheim, A. SUPPORT Tools for evidence-informed health Policymaking (STP) 13: Preparing and using policy briefs to support evidence-informed policymaking. *Health Research Policy and Systems* **7**, 1-9 (2009).

13 Dobbins, M., Jack, S., Thomas, H. & Kothari, A. Public health decision-makers' informational needs and preferences for receiving research evidence. *Worldviews Evid Based Nurs* **4**, 156-163, doi:10.1111/j.1741-6787.2007.00089.x (2007).

14 Innvaer, S., Vist, G., Trommald, M. & Oxman, A. Health policy-makers' perceptions of their use of evidence: a systematic review. *Journal of health services research & policy* **7**, 239-244 (2002).

15 Jessani, N., Kennedy, C. & Bennett, S. Enhancing evidence-informed decision making: strategies for engagement between public health faculty and policymakers in Kenya. *Evid Policy* **13**, 225-253 (2016).

16 Hyder, A. A. *et al.* National policy-makers speak out: are researchers giving them what they need? *Health Policy and Planning* **26**, 73-82, doi:10.1093/heapol/czq020 (2010).

17 Mahendradhata, Y. & Kalbarczyk, A. Prioritizing knowledge translation in low- and middle-income countries to support pandemic response and preparedness. *Health Res Policy Syst* **19**, 5, doi:10.1186/s12961-020-00670-1 (2021).

For peer review only

18 Mbogning Fonkou, M. D. *et al.* COVID-19 Pandemic Related Research in Africa: Bibliometric Analysis of Scholarly Output, Collaborations and Scientific Leadership. *Int J Environ Res Public Health* **18**, doi:10.3390/ijerph18147273 (2021).

19 Kana, M. A., LaPorte, R. & Jaye, A. Africa's contribution to the science of the COVID-19/SARS-CoV-2 pandemic. *BMJ Global Health* **6**, e004059, doi:10.1136/bmjgh-2020-004059 (2021).

20 Guleid, F. H. *et al.* A bibliometric analysis of COVID-19 research in Africa. *BMJ Glob Health* **6**, doi:10.1136/bmjgh-2021-005690 (2021).

21 Polit, D. F. & Beck, C. T. Generalization in quantitative and qualitative research: Myths and strategies. *International journal of nursing studies* **47**, 1451-1458 (2010).

Figures

Figure 1: Framework for assessing the usefulness of EEVIDEM activities

For peer review only

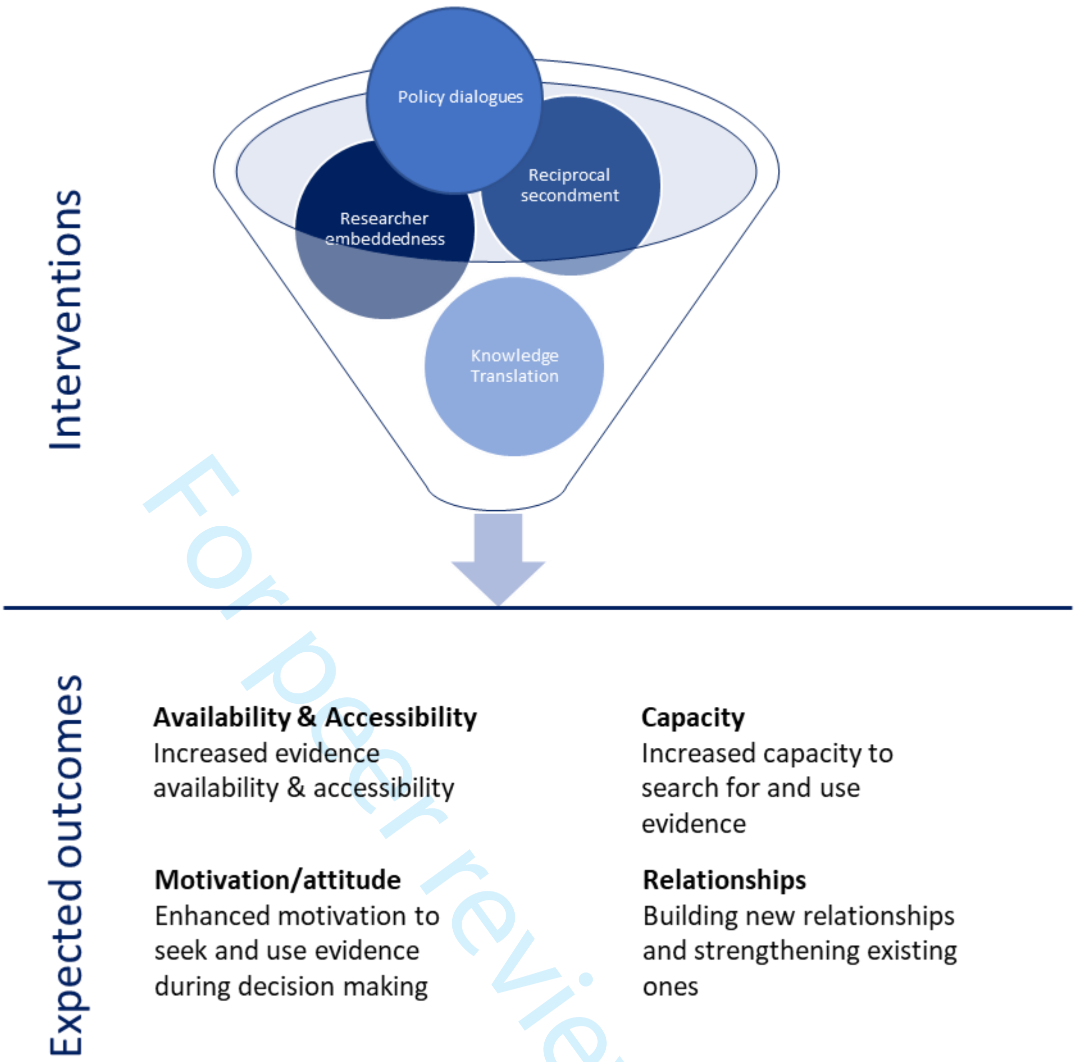

Figure 1: Framework for assessing the usefulness of EEVIDEM activities

For peer review only

COREQ (COnsolidated criteria for REporting Qualitative research) Checklist

A checklist of items that should be included in reports of qualitative research. You must report the page number in your manuscript where you consider each of the items listed in this checklist. If you have not included this information, either revise your manuscript accordingly before submitting or note N/A.

Topic	Item No.	Guide Questions/Description	Reported on Page No.
**Domain 1: Research team and reflexivity**			
Personal characteristics			
Interviewer/facilitator	1	Which author/s conducted the interview or focus group?	
Credentials	2	What were the researcher's credentials? E.g. PhD, MD	
Occupation	3	What was their occupation at the time of the study?	
Gender	4	Was the researcher male or female?	
Experience and training	5	What experience or training did the researcher have?	
Relationship with participants			
Relationship established	6	Was a relationship established prior to study commencement?	
Participant knowledge of the interviewer	7	What did the participants know about the researcher? e.g. personal goals, reasons for doing the research	
Interviewer characteristics	8	What characteristics were reported about the inter viewer/facilitator? e.g. Bias, assumptions, reasons and interests in the research topic	
**Domain 2: Study design**			
Theoretical framework			
Methodological orientation and Theory	9	What methodological orientation was stated to underpin the study? e.g. grounded theory, discourse analysis, ethnography, phenomenology, content analysis	
Participant selection			
Sampling	10	How were participants selected? e.g. purposive, convenience, consecutive, snowball	
Method of approach	11	How were participants approached? e.g. face-to-face, telephone, mail, email	
Sample size	12	How many participants were in the study?	
Non-participation	13	How many people refused to participate or dropped out? Reasons?	
Setting			
Setting of data collection	14	Where was the data collected? e.g. home, clinic, workplace	
Presence of non-participants	15	Was anyone else present besides the participants and researchers?	
Description of sample	16	What are the important characteristics of the sample? e.g. demographic data, date	
Data collection			
Interview guide	17	Were questions, prompts, guides provided by the authors? Was it pilot tested?	
Repeat interviews	18	Were repeat inter views carried out? If yes, how many?	
Audio/visual recording	19	Did the research use audio or visual recording to collect the data?	
Field notes	20	Were field notes made during and/or after the inter view or focus group?	
Duration	21	What was the duration of the inter views or focus group?	
Data saturation	22	Was data saturation discussed?	
Transcripts returned	23	Were transcripts returned to participants for comment and/or	

For peer review only

Topic	Item No.	Guide Questions/Description	Reported on Page No.
		correction?	
**Domain 3: analysis and findings**			
Data analysis			
Number of data coders	24	How many data coders coded the data?	
Description of the coding tree	25	Did authors provide a description of the coding tree?	
Derivation of themes	26	Were themes identified in advance or derived from the data?	
Software	27	What software, if applicable, was used to manage the data?	
Participant checking	28	Did participants provide feedback on the findings?	
Reporting			
Quotations presented	29	Were participant quotations presented to illustrate the themes/findings? Was each quotation identified? e.g. participant number	
Data and findings consistent	30	Was there consistency between the data presented and the findings?	
Clarity of major themes	31	Were major themes clearly presented in the findings?	
Clarity of minor themes	32	Is there a description of diverse cases or discussion of minor themes?	

Developed from: Tong A, Sainsbury P, Craig J. Consolidated criteria for reporting qualitative research (COREQ): a 32-item checklist for interviews and focus groups. *International Journal for Quality in Health Care*. 2007. Volume 19, Number 6: pp. 349 – 357

Once you have completed this checklist, please save a copy and upload it as part of your submission. DO NOT include this checklist as part of the main manuscript document. It must be uploaded as a separate file.

For peer review only
